# Deconvoluting thermomechanical effects in X-ray diffraction data using machine learning

**DOI:** 10.1107/S2053273325000403

**Published:** 2025-01-31

**Authors:** Rachel E. Lim, Shun-Li Shang, Chihpin Chuang, Thien Q. Phan, Zi-Kui Liu, Darren C. Pagan

**Affiliations:** a Pennsylvania State University University Park PA 16802 USA; b Lawrence Livermore National Laboratory,Livermore CA 94550 USA; c Argonne National Laboratory, Lemont, IL 60439, USA; Institute of Crystallography - CNR, Bari, Italy

**Keywords:** strain, synchrotron X-ray diffraction, first-principles calculations, stress, Gaussian process regression, superalloys, machine learning, physics-based modeling

## Abstract

X-ray diffraction is ideal for probing the sub-surface state during complex or rapid thermomechanical loading of crystalline materials. However, challenges arise as the size of diffraction volumes increases due to spatial broadening and because of the inability to deconvolute the effects of different lattice deformation mechanisms. Here, we present a novel approach that uses combinations of physics-based modeling and machine learning to deconvolve thermal and mechanical elastic strains for diffraction data analysis. The method builds on a previous effort to extract thermal strain distribution information from diffraction data. The new approach is applied to extract the evolution of the thermo­mechanical state during laser melting of an Inconel 625 wall specimen which produces significant residual stress upon cooling. A combination of heat transfer and fluid flow, elasto-plasticity and X-ray diffraction simulations is used to generate training data for machine-learning (Gaussian process regression, GPR) models that map diffracted intensity distributions to underlying thermomechanical strain fields. First-principles density functional theory is used to determine accurate temperature-dependent thermal expansion and elastic stiffness used for elasto-plasticity modeling. The trained GPR models are found to be capable of deconvoluting the effects of thermal and mechanical strains, in addition to providing information about underlying strain distributions, even from complex diffraction patterns with irregularly shaped peaks.

## Abstract 2

Bayesian machine learning, trained by physics-based simulations, is used to separate the effects of temperature and mechanical loading within *in situ* diffraction data.

## 1. Introduction

Understanding and controlling the development of residual stress during traditional welding, and now additive manufacturing (AM), is an ongoing challenge. Rapid, localized heating then solidification leads to large thermal stresses which, in turn, lead to plastic flow and residual stress. These residual stresses can drive hot-cracking or cause such severe distortions that a part is unusable. Optical and thermography measurements provide a means to characterize the temperature gradients driving stress development (Moylan *et al.*, 2014 ▸; Everton *et al.*, 2016 ▸; Fox *et al.*, 2017 ▸; Fisher *et al.*, 2018 ▸; Montazeri *et al.*, 2019 ▸; Dunbar & Nassar, 2018 ▸; Forien *et al.*, 2020 ▸; Ashby *et al.*, 2022 ▸), but are limited to the surface. In addition, while significant focus has been placed on developing high-speed X-ray imaging at synchrotron sources to monitor melt pool characteristics and the development of porosity in support of AM processing, the use of scattering and the study of stress development have been limited in comparison. However, recent work is building upon imaging efforts to also use scattering to study complex rapid solidification processes (Kenel *et al.*, 2016 ▸; Calta *et al.*, 2018 ▸; Cunningham *et al.*, 2019 ▸; Hocine *et al.*, 2020 ▸; Oh *et al.*, 2021*a* ▸; Oh *et al.*, 2021*b* ▸; Thampy *et al.*, 2020 ▸; Silveira *et al.*, 2023 ▸; Chen *et al.*, 2023 ▸; Scheel *et al.*, 2023 ▸; Dass *et al.*, 2023 ▸). More specifically, diffraction processes are capable of monitoring the development of texture, phases and residual strains (and related stress).

An acute challenge in using diffraction to monitor stress development in combined thermal and mechanical loading is that both temperature and mechanical stress alter the state of the crystal lattice (which is what is probed during diffraction measurements). Specifically, in cubic crystal types, both temperature and hydrostatic stress will alter the volumetric portion of the lattice strain tensor in the same fashion, making deconvolution of the effects difficult. Assumptions can be made regarding whether the thermal or mechanical alterations to the lattice state are dominant for analysis, but these can introduce significant uncertainty into interpretation of data when magnitudes become approximately equal. In addition, the spread of thermal and mechanical strain distributions through a diffraction volume can be extensive, particularly when using high-energy X-rays in transmission through thick specimens. A single diffraction image (projection) does not provide sufficient information for direct reconstruction of the distributions present.

To overcome these challenges, we present a machine learning (ML) approach in which Gaussian process regression (GPR) models are trained to learn the relationship between diffraction patterns underlying thermomechanical strain distributions. The ML training process is supported by physics-based modeling of the heating and cooling processes that lead to stress development. This modeling, along with accurate diffraction simulations, is used to create a ‘dictionary’ of diffraction patterns for ML model training. The trained ML model is then transferred to the analysis of experimental data. To attain accurate thermal and mechanical properties, density functional theory (DFT) is utilized. This work builds on our previous effort to extract temperature (thermal strain) distribution metrics from *in situ* diffraction patterns (Lim *et al.*, 2023 ▸). However, as mentioned, due to convolution of the effects of thermal and mechanical strain, this previous effort was only accurate at high temperatures. Transfer learning is particularly appropriate for cases where the underlying physics is fairly well understood and modeled. Any uncertainty in the accuracy of the model may have implications for ‘transferring’ to real data. Thus, high-fidelity models are required and improvements to our previous work have been made through the following advances: (i) the use of DFT for temperature-dependent thermomechanical properties, (ii) modeling of stress distributions using a thermomechanical elasto-plasticity model, (iii) update of the X-ray diffraction modeling framework to incorporate anisotropic lattice strains during mechanical loading, and (iv) update of the GPR model training to utilize anisotropic diffraction ring expansion and contraction.

**Figure 1:**
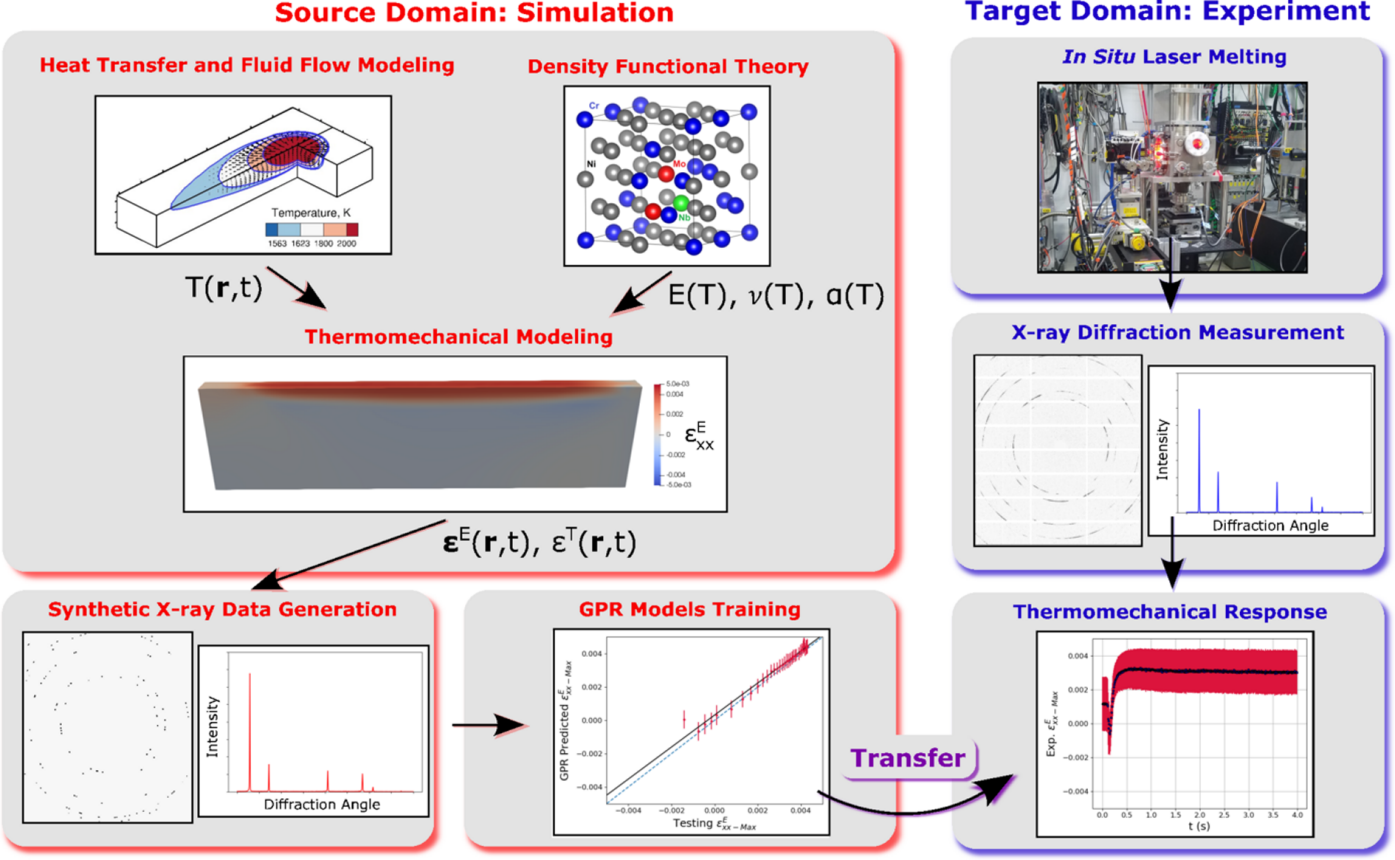
Overview of the simulations used to train Gaussian process regression (GPR) models for X-ray diffraction data analysis. The trained models are subsequently transferred to the target (experimental) domain to separate thermal and mechanical strain effects from experimental X-ray diffraction data. Temperature fields 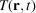 and temperature-dependent properties [Young’s modulus 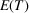, Poisson’s ratio 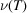 and coefficient of thermal expansion 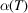] are used as input for thermomechanical modeling to predict evolving thermal 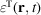 and elastic strains 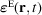. These strains are used for input into X-ray diffraction simulations and GPR model training. The trained GPR models are then used for analysis of experimental data.

The approach is demonstrated through extraction of thermal and elastic strain distribution metrics from a wall specimen of Inconel 625 in a transmission geometry that has become a standard for *in situ* AM experiments. An overview of the data processing workflow is provided in Fig. 1 ▸. This work begins by describing the experimental data to be analyzed in Section 2. Next, in Section 3, summaries of the various physics models used for GPR model training are provided. The GPR models used and their training are described in Section 4. The trained GPR models are then applied to experimental *in situ* diffraction data in Section 5. The article ends by discussing the approach and avenues for future study in Section 6.

## 2. Material and experiment description

The material tested was Inconel 625 (IN625) which was extracted from a block built using laser powder bed fusion at the National Institute of Standards and Technology (NIST) (Levine *et al.*, 2020 ▸). The block was built in an EOS M290 system using powder also obtained from EOS. The block dimensions were 50 × 15 × 6 mm, where the 6 mm direction is the build direction. The build followed manufacturer recommendations (Son *et al.*, 2020 ▸) of laser power of 285 W, laser speed of 960 mm s^−1^ and interlayer rotation of 67.5°. After build, the specimen was stress-relief heat treated at 800°C for 2 h. A wall specimen was then extracted using electro-discharge machining with dimensions of 15 × 0.53 × 3 mm, with the 3 mm dimension being aligned with the build dimension. Previous characterization (including electron backscatter diffraction) of the material found that the grain size ranged from approximately 10 to 100 µm with a mean between 25 and 30 µm (Lim *et al.*, 2023 ▸).

**Figure 2:**
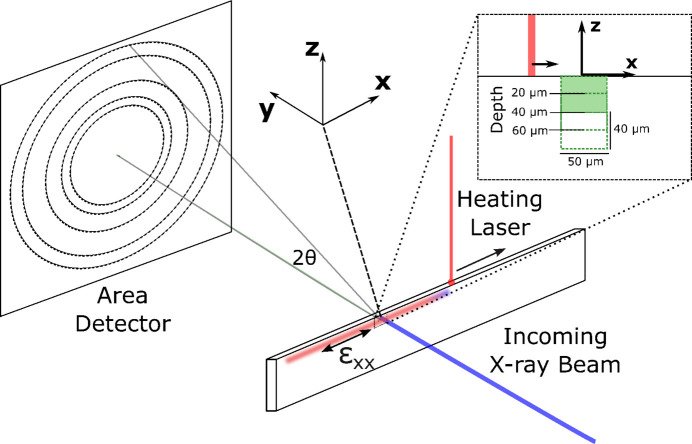
Schematic of the (simulation and experiment) geometry for the wall laser melting experiment. The inset indicates points where diffraction measurements and simulations were performed with respect to the top of the sample. The X-ray beam was centered 20 µm below the sample surface during the experiment (shaded green), while the X-ray beam was centered 20, 40 and 60 µm below the sample surface for various simulations used in GPR model training.

The wall specimen was then used for an *in situ* laser melting experiment at Sector 1-ID of the Advanced Photon Source. An existing *in operando* laser powder bed fusion (LPBF) simulator (Zhao *et al.*, 2017 ▸) consisting of a laser and control, sealed chamber and sample staging was used for laser melting. A schematic of the experimental geometry is provided in Fig. 2 ▸. The laser system was composed of a ytterbium fiber laser (IPG YLR-500-AC) controlled with an intelliSCAN_de_ 30. Using this system, the laser was rastered along the top of the wall specimen in the *x* direction at velocity *v* with magnitude of 0.05 m s^−1^ and power *P* of 120 W (2400 J m^−1^), generating a relatively large high-temperature region and resulting residual stresses. During laser melting, a primarily uniaxial tensile residual stress developed along the top of the wall specimen. This process consisted of (i) rapid expansion during heating, (ii) compressive stresses sufficiently high to generate plastic flow and permanent compression, and (iii) extension and tensile loading as the specimen cooled to accommodate compatibility. The primary tensile mechanical (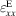) strains that develop during cooling are also illustrated on the specimen in Fig. 2 ▸.

**Figure 3:**
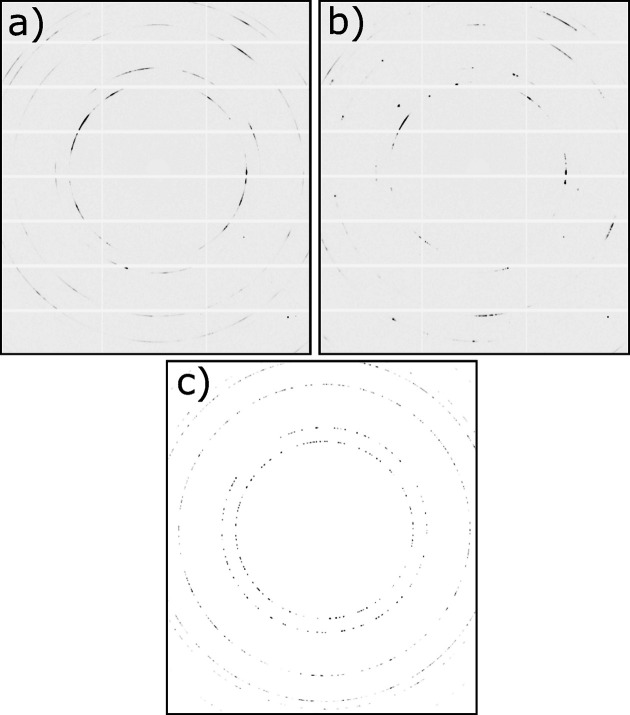
Representative diffraction images from (*a*) the experimental sample before laser melting, (*b*) the experimental sample after laser melting, (*c*) a thermomechanical simulation used for GPR surrogate model training. Maximum intensity thresholds have been selected for each image to make the diffraction peaks and their extent visible.

During the laser melting, the wall specimen was illuminated by a 61.332 keV X-ray beam (Yb *K*α) with dimensions of 50 µm (along **x**) × 30 µm (along **z**). The energy divergence of the beam was 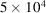. The diffraction data were collected using a Pilatus3 X CdTe 2M detector sitting 752 mm downstream of the sample. The beam center was placed 20 µm below the top of the specimen and the sample and beam were fixed as the laser passed over the diffraction volume. The detector was positioned such that five complete (111, 200, 220, 311, 222) and one nearly complete (400) diffraction peaks were measured on the detector (maximum 2θ angle of 13°). Diffraction data were collected for 4 s at a rate of 250 Hz and exposure time of 1 ms, with data collection synchronized such that the laser passed over the diffraction volume at 0.2 ms. Representative diffraction images collected before and after laser melting are shown in Figs. 3 ▸(*a*) and 3 ▸(*b*), respectively. We note that, with the rapid data collection rate, minor phases such as the δ phase do not diffract sufficient intensity to be characterized.

**Figure 4:**
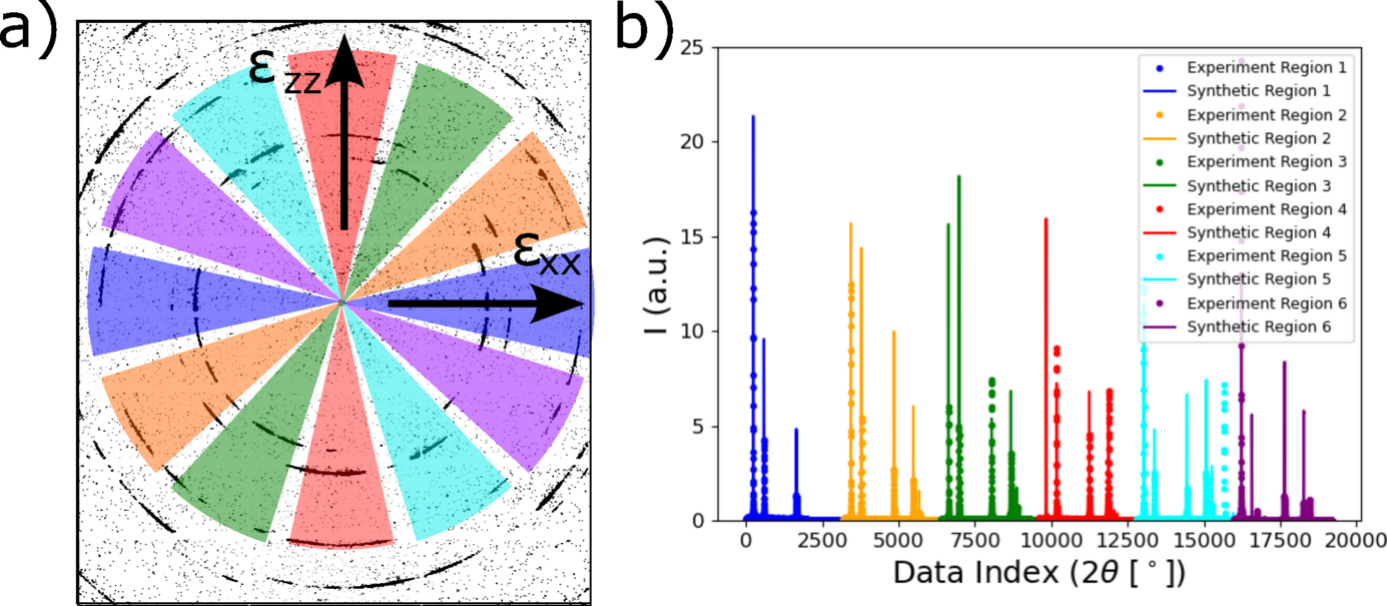
(*a*) Illustration of the six sets of azimuthal bins (colored) around which diffraction images were integrated. (*b*) Concatenation of the 1D intensity data from the different bins for use in ML models. A comparison of representative experimental and simulated diffraction data is shown.

For use in ML models (described below), each diffraction image was then integrated into six different azimuthal bins, which are illustrated in Fig. 4 ▸(*a*). The bins (blue) along the image horizontal are aligned with projections of the strain tensor 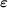 near the *x* direction (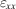), while vertical bins (red) are projections near the *z* direction (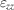). The other four sets of bins (orange, green, teal, purple) further capture the anisotropy of the diffraction ring evolution. Once integrated, the six sets of 1D diffraction profiles were concatenated into a single vector, illustrated in Fig. 4 ▸(*b*). This choice of binning strategy was made as a compromise between probing a sufficient number of grains along different directions while still capturing the anisotropy of diffraction ring shape changes due to multiaxial elastic strains.

## 3. Training data generation

### 3.1. Heat transfer and fluid flow

The heat transfer and fluid flow modeling that is used to generate input temperature fields is discussed in detail by Mukherjee *et al.* (2018*a* ▸, 2018*b* ▸). The model is designed to be capable of simulating the LPBF process, but modeling of melting of solid materials is also possible. The code uses a finite difference scheme to simultaneously solve for conservation of mass, energy and momentum. An adaptive grid is used with the calculation grid refining around the current position of the laser spot and melt pool (*i.e.* the regions with largest thermal gradients and the melt pool interface). The conditions and material parameters mirror those found in the previous effort upon which this work builds (Lim *et al.*, 2023 ▸), with the only modification being that the simulations were re-performed with an extended cooldown period (5 s) to allow the specimen to cool closer to room temperature for more input into the thermomechanical processing. The material properties used for simulations were calculated using *JMatPro* (Saunders *et al.*, 2003 ▸) and are summarized in Table 1 ▸.

A series of simulations were performed rastering a simulated laser heat source over the top of wall specimens with the same thickness (0.53 mm) and height (3 mm) as in the experiment. The length of the specimen was 11.5 mm to reduce computational cost. The heat source moved along the top surface (normal *z*) in the *x* direction. Following Lim *et al.* (2023 ▸), nine different simulations were performed around the nominal experimental laser setting (power and speed varied) with the same approximate laser size and they are summarized in Table 2 ▸. The extra thermomechanical simulations beyond 120 W and 0.04 m s^−1^ help to compensate for any uncertainty in the laser parameters applied during the experiment. After the heat source passed over the length of the specimen, the sample was allowed to cool for 5 s, as mentioned. The primary outputs are temperature fields *T*(**r**), where **r** denotes position, saved at a rate of 500 Hz as the heat source moved and then 100 Hz as the sample cooled for a total of 150 time steps. As mentioned, the code adaptively changes the calculation grid throughout the simulation for computational efficiency. To prepare the temperature fields for use in the next steps of the workflow (Fig. 1 ▸), the simulations were post-processed using bilinear interpolation to remap the output to a regular grid with a point spacing of 20 µm.

### 3.2. Thermomechanical model

Once the nine time series temperature fields were calculated from the heat transfer and fluid flow modeling, they were used as external data inputs for an elasto-plasticity model in *ANSYS* (ANSYS, 2011 ▸) to generate thermal 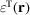 and elastic strain 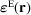 fields. The element type used was eight node ‘brick’ elements with three degrees of freedom (translations in *x*, *y* and *z*) at each node. Element sizes ranged from 125 to 140 µm (approximately 10000 elements per simulation) along their edges.

**Figure 5:**
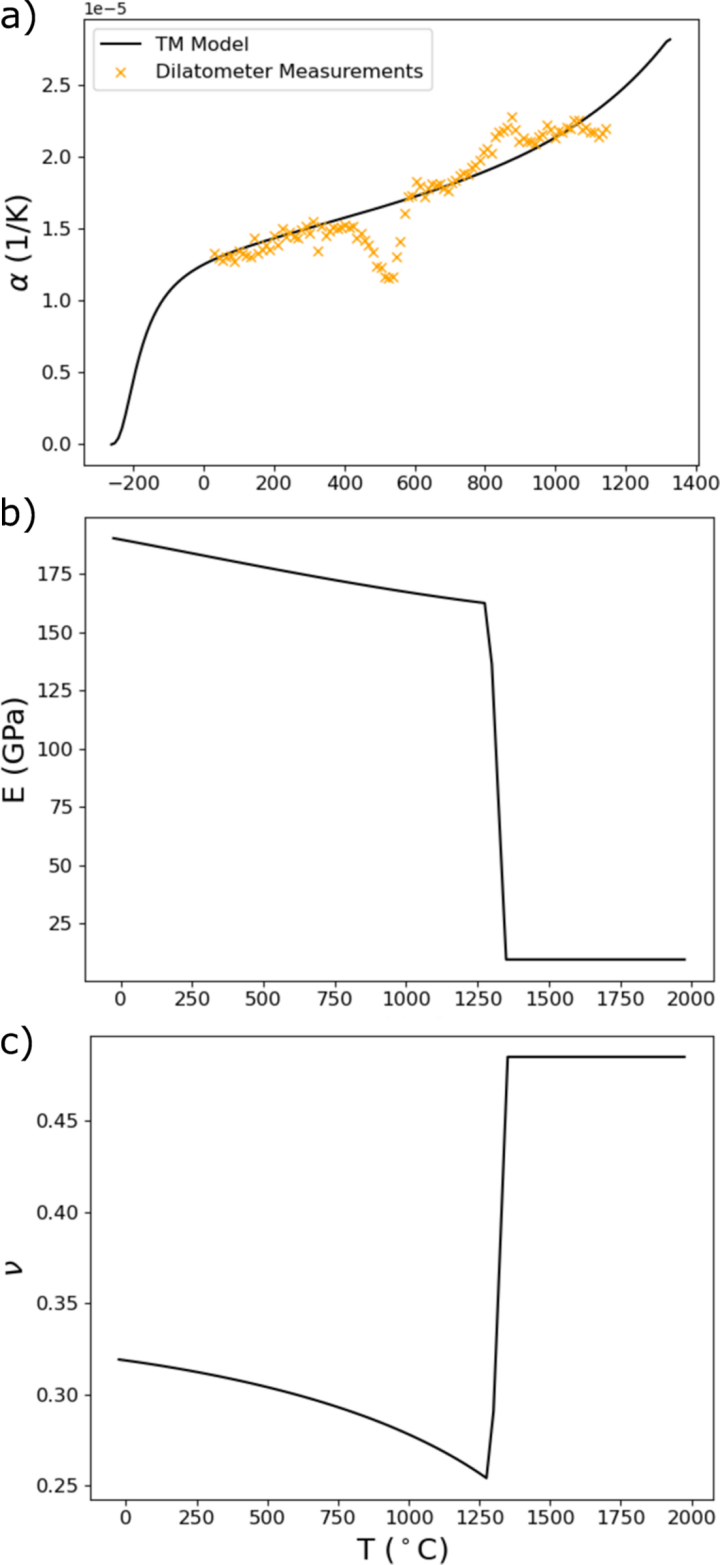
Temperature-dependent (*a*) coefficient of thermal expansion α, (*b*) isotropic Young’s modulus *E* and (*c*) isotropic Poisson’s ratio ν used as input for the finite element simulations of the development of thermal and residual stresses in the AM IN625 wall specimens derived from the single-crystal elastic moduli calculated from DFT. Dilatometer measurements of thermal expansion used to evaluate the DFT results are also provided in (*a*).

In the model used (Taylor *et al.*, 1970 ▸; ANSYS, 2011 ▸), strains are additively decomposed into thermal, elastic and plastic portions with the total deformation field being that required to maintain compatibility,
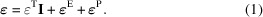
The thermomechanical model employed includes thermal expansion, 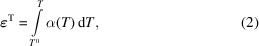
and temperature-dependent isotropic linear elasticity,
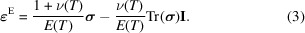
Plasticity is governed by rate-independent J2 plasticity and linear hardening where yielding occurs when 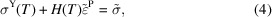
where 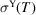 is the initial temperature-dependent yield strength, 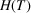 is the temperature-dependent hardening rate, 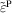 is the equivalent plastic strain and 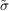 is the equivalent (von Mises) stress. If the stresses are sufficient to initiate yielding (and ultimately the development of residual stress), plastic flow occurs. As the literature is limited regarding temperature-dependent properties of specific alloy compositions, the temperature-dependent coefficient of thermal expansion 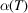 and the elastic moduli, Young’s modulus 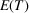 and Poisson’s ratio 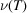 were determined using first-principles DFT. The process by which these values were calculated is presented in Appendix *A*[App appa] (Section *A*1). The thermal expansion and elastic moduli used for thermomechanical model input are provided in Fig. 5 ▸. The temperature-dependent coefficient of thermal expansion measured from the same IN625 build as the experimental thin wall with dilatometry and used to evaluate the DFT results is also provided in Fig. 5 ▸(*a*). The tabulated yield and hardening parameters were determined from a combination of mechanical tests (≤500°C) performed on the same material and IN625 parameters built into the *ANSYS* package (Table 3 ▸).

**Figure 6:**
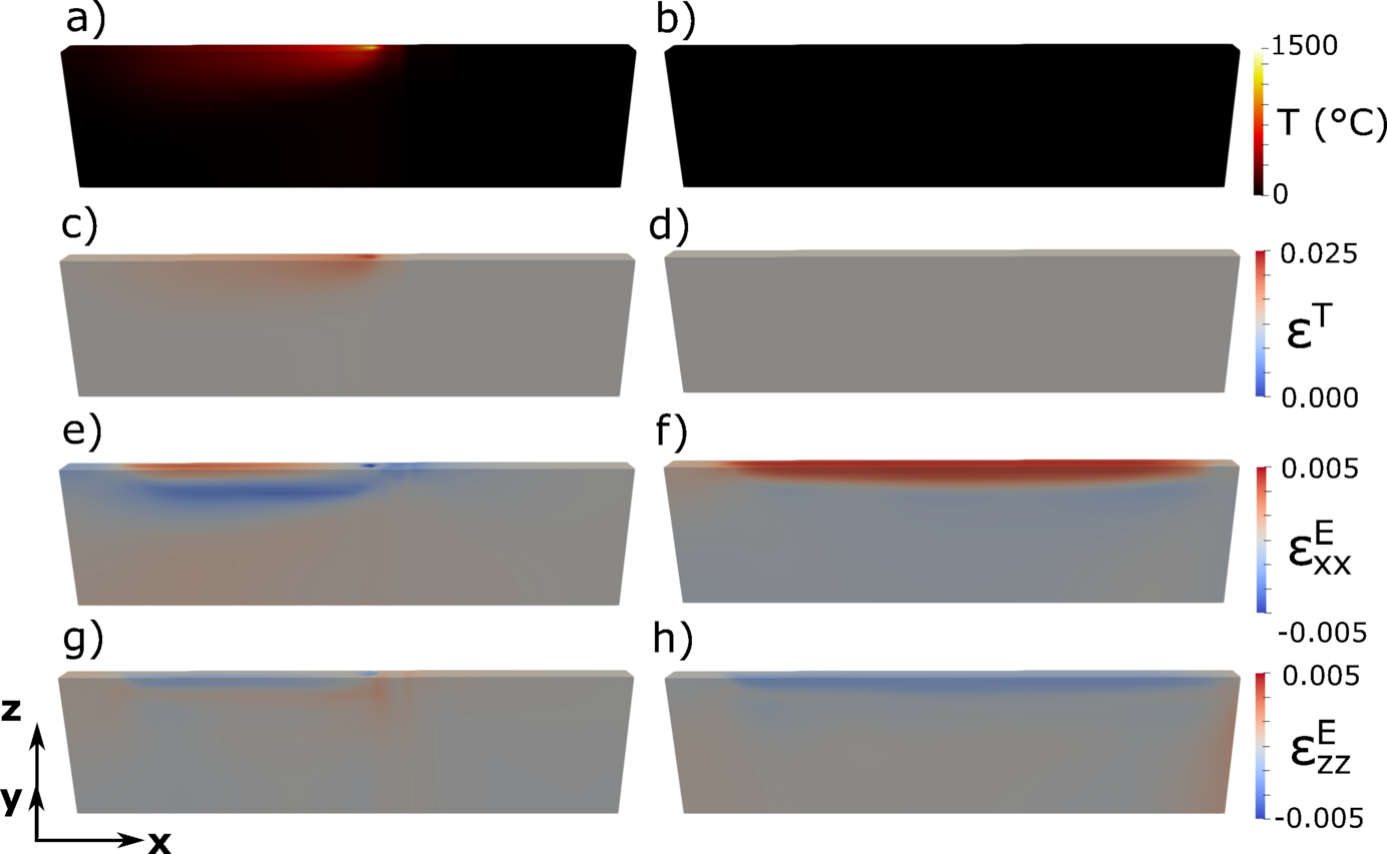
Representative fields from the thermomechanical simulations (*P* = 120 W, *v* = 0.05 m s^−1^ and spot diameter of 100 µm) as the laser is passing overhead: (*a*) *T*, (*c*) 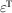, (*e*) 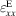 and (*g*) 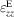. Representative fields from the same laser parameter simulation after cooling: (*b*) *T*, (*d*) 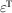, (*f*) 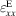 and (*h*) 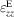.

Each time step takes the previously calculated thermal field and calculates local thermal strains [equation (2)] from the temperature-dependent coefficient of thermal expansion previously calculated using DFT (Shang *et al.*, 2024 ▸). A strain field is then calculated which satisfies both compatibility (in conjunction with the thermal and plastic strain) and mechanical equilibrium. In all thermomechanical simulations, the thermal gradient was sufficient to initiate yielding and plastic flow. While plastic strain does not directly alter the crystal lattice and resulting diffraction, the deformation incompatibility created by the plastic strain gives rise to elastic strain and stress distributions which are measurable through diffraction. Fig. 6 ▸ shows representative fields (*P* = 120 W, *v* = 0.05 m s^−1^ and spot diameter of 100 µm) calculated by the thermomechanical model as the laser passes over the sample [Figs. 6 ▸(*a*), 6 ▸(*c*), 6 ▸(*e*) and 6 ▸(*g*)] and after cooling [Figs. 6 ▸(*b*), 6 ▸(*d*), 6 ▸(*f*) and 6 ▸(*h*)]. The thermal and elastic strains are plotted with different color scales due to the large differences in maximum magnitude. Important to note are the relatively large residual tensile 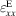 strains that develop during the cooling process.

To facilitate X-ray diffraction simulations from the elasto-plasticity results, a series of scripts were developed to convert *ANSYS* mechanical data into a format compatible with X-ray simulations. This effort included developing (i) a new Python-based binding for ASCII output of *ANSYS* data; (ii) a script to convert element position, stress and strain data into the coordinate system used by the diffraction simulation framework; and (iii) developing a framework to interpolate thermomechanical data onto a finer grid (20 µm spacing) necessary for diffraction simulations.

### 3.3. X-ray diffraction simulations

The forward modeling framework for generating synthetic diffraction patterns for model training utilizes *HEXRD* (Bernier *et al.*, 2011 ▸; Nygren *et al.*, 2020 ▸) and is described in more detail by Pagan *et al.* (2020 ▸). Simulations build on our previous effort (Lim *et al.*, 2023 ▸), but have now been modified to include the anisotropic scattering effects of the deviatoric portion of the elastic strain tensor on the diffraction simulations. The series of nine elastic strain 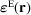 and thermal strain fields 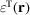 (remapped to a 20 µm grid) calculated from thermo­mechanical simulations were used as input for the diffraction simulations. A scattering volume is built around each field point in which discrete scattering crystals can be placed. Here, grains are modeled with a 25 µm equivalent grain size so two crystals are inserted around each scattering volume for over 200 crystals in the diffraction volume. Each crystal is modeled with 1° of misorientation to provide a finite peak width perpendicular to the radial direction on the detector and ease numeric issues associated with calculating the diffraction condition. The crystals are randomly oriented which is consistent with the lack of texture found in the heat-treated IN625 being modeled. Here we project the macroscopic elastic and thermal strains onto the discrete diffracting crystals.

To model the effects of thermal and elastic strains, the reciprocal-lattice vectors, **g**, within each grain are stretched from a reference 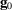: 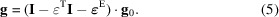
The reference cubic lattice parameter used for calculation of 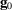 was 3.5981 Å. At each time step, diffraction from a set of lattice planes within a grain is determined to occur based on the incoming X-ray energy and bandwidth, 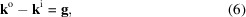
where 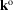 and 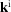 are the outgoing and incoming wavevectors, respectively. The list of reciprocal-lattice vectors (lattice planes) checked had a maximum 2θ of 13°, matching the experiment. The diffraction events are then projected to the detector. Here the instrument geometry and detector were selected to match the experiment. If the temperature within a scattering volume exceeds the solidus temperature, no diffraction event is projected. Diffraction events from different sets of lattice planes are weighted by the structure factor. Drops in intensity due to absorption are neglected as this effect is minimal at high energy. Here, we emphasize that as the reciprocal-lattice vectors in grains modeled at different positions are stretched by varying amounts based on the thermal and elastic strain fields, both diffraction peak shifts and broadening are naturally captured. Specifically, we capture broadening of a diffraction peak from distributions of temperature and mechanical strain in the diffraction volume, broadening from the relatively large diffraction volume size and broadening from the finite-energy bandwidth (see Pagan *et al.*, 2020 ▸). We do not specifically incorporate broadening from grain-to-grain strain interactions from elastic anisotropy or dislocation broadening as plastic strain accumulates. In this case, the extreme thermomechanical distributions present in the diffraction volume are assumed to be the dominant source of broadening. The modeled broadening is a key feature for the ML model to learn with regards to the distributions of strain present in the diffraction volume.

Diffraction simulations for each elasto-plasticity simulation were repeated three times with the beam placed at 20, 40 and 60 µm from the top of the specimen and the rastering laser beam (see Fig. 2 ▸). Note that the experiment was only performed with the beam centered at 20 µm from the sample surface. The purpose of this is twofold. Performing simulations at different X-ray beam positions on the sample increased the amount of diffraction images generated for model training, and it helped to account for any uncertainty in beam placement with respect to the temperature field in the sample. In total, 27 sets of X-ray simulations in conditions similar to the experiment (three different diffraction volumes for each of the nine laser conditions simulated) were performed, with 75 diffraction images from each. This produced 2025 diffraction images in total for GPR model training. Once the 2D diffraction patterns were simulated for the entire time series, each pattern was integrated azimuthally in the same fashion as the experimental data, which is illustrated in Fig. 4 ▸. Note that the mismatch between synthetic and experimental data of the relative intensities of each peak within each color highlights the local variations of texture due to the combination of beam and grain size. After integration, background scattering was added to the same data of the same magnitude as that observed in the experiment.

## 4. ML model and training

GPR (Williams & Rasmussen, 2006 ▸) was used for learning the mapping between diffraction data and the distributions of thermal and elastic strain present within the diffraction volume. The approach here is an extension of our previous effort of mapping isotropic diffraction ring evolution to temperature (thermal strain) fields present, to now map anisotropic diffraction ring evolution (both peak shifts and broadening) to elastic and thermal strain distributions present. GPR assumes a normal distribution of mapping functions, *f* (here strain field metrics), from input data, **x** (here diffracted intensity distributions along different sample directions). The mean mapping function (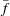) from the normal distribution is the prediction of the model. A natural benefit of the approach is that uncertainty is estimated from the variance of the mapping functions. Other ML approaches, such as neural networks, do not as readily provide measures of the uncertainty of predictions. The mapping functions learned are linear combinations of input training data 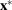, where the coefficients are dependent on the similarity of the training and input data. If input data are similar (here determined by Euclidean distance) to training data, those training data are more heavily weighted, as dictated by the chosen covariance function (see below). In addition, if the input data for the model are not similar to any training data, the uncertainty of the model prediction increases.

For model training, we use the rational quadratic covariance function (kernel) *k* as opposed to the more common exponentiated quadratic kernel. The rational quadratic kernel is given as 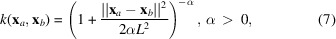
where 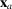 and 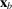 are two input data points, while α and *L* control the decay rate for weights. As α decreases, training data of increased dissimilarity from the input data are incorporated into model predictions, while *L* provides a further control if necessary. Here, we use *L* = 1 and α = 1. The choice of the rational quadratic kernel slows the decay of the interpolation, leading to more training data points being used in each prediction.

For GPR model training and testing, 26 of the data sets were used, while one (No. 13, see Table 2 ▸) was reserved for testing. We trained 16 different GPR models to learn mapping between the mean, maximum, minimum and standard deviations (STDs) of 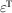, 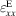, 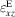 and 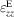 within the illuminated diffraction volume and the diffraction data. For these simulations (and the experiment), 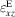 is minimal across the sample; however, this still provides a further test of the model. In this diffraction geometry, the projections of **g** along **y** are minimal and as such 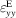, 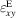 and 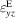 are not probed. We note that the trained GPR models are not fitting analytic functions to the peaks, and thus not restricted to regularly shaped diffraction peaks (*e.g.* Gaussian or Lorentzian) and are capable of mapping ‘split’ peaks or those with long tails to underlying strain distributions (Lim *et al.*, 2023 ▸).

**Figure 7:**
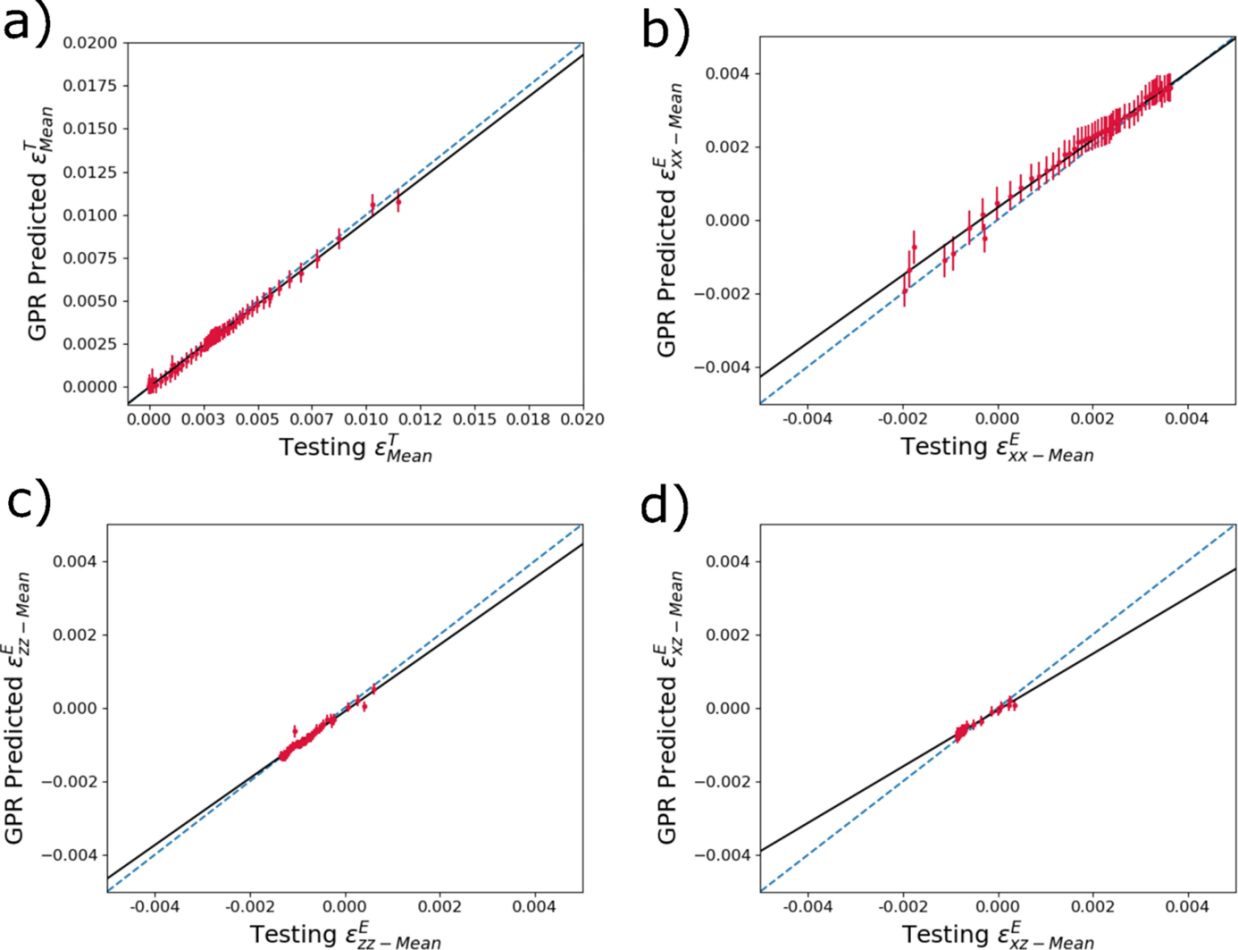
Accuracy predicting reserved testing data of trained surrogate models to determine the mean of (*a*) 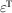, (*b*) 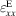, (*c*) 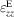 and (*d*) 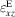 within a diffraction volume from input diffraction spectra. The dashed blue line denotes one-to-one correlation, and the black line is a linear fit.

To examine the accuracy of the predictions of the trained GPR models, the reserved testing data set was used as input for the trained models and the output predictions were then compared with the true thermomechanical distribution quantities. Fig. 7 ▸ shows the comparisons between mean strains in diffraction volumes from the thermomechanical model output (here serving as ground truth) and predictions from the trained GPR model using diffraction data as input. Figs. 7 ▸(*a*), 7 ▸(*b*), 7 ▸(*c*) and 7 ▸(*d*) correspond to 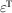, 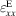, 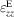 and 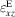. Note the difference in strain scales between thermal and elastic strains (due to large differences in magnitude) which will continue through the rest of the work. In general, the GPR model predictions for mean strains are accurate with the ground truth falling within the uncertainty bounds. In Fig. 7 ▸(*a*), the thermal strain 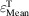 predictions tend to be closer to the ground truth at room temperature, with deviations at higher strains (temperatures). For the primary mechanical strains 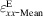 in Fig. 7 ▸(*b*), the opposite is true: the accuracy tends to be better at larger strains. The predictions of the mean 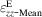 and 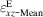 generally fit well within the strain bounds where there are data (besides a small number of outliers), but we note that the magnitude of mean strains is relatively low for these strain components.

**Figure 8:**
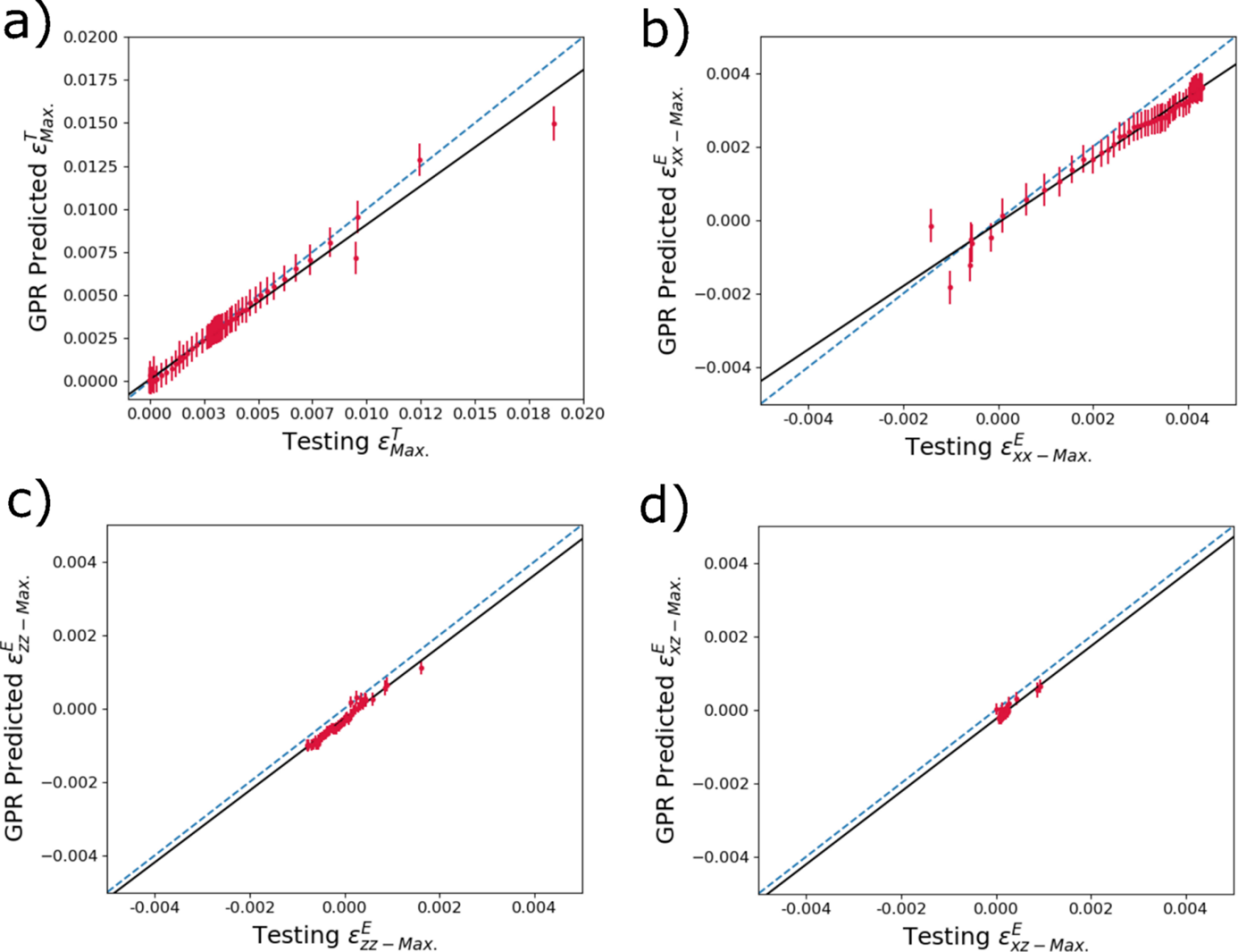
Accuracy predicting reserved testing data of trained surrogate models to determine the maximum of (*a*) 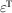, (*b*) 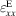, (*c*) 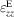 and (*d*) 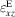 within a diffraction volume from input diffraction spectra. The dashed blue line denotes one-to-one correlation, and the black line is a linear fit.

**Figure 9:**
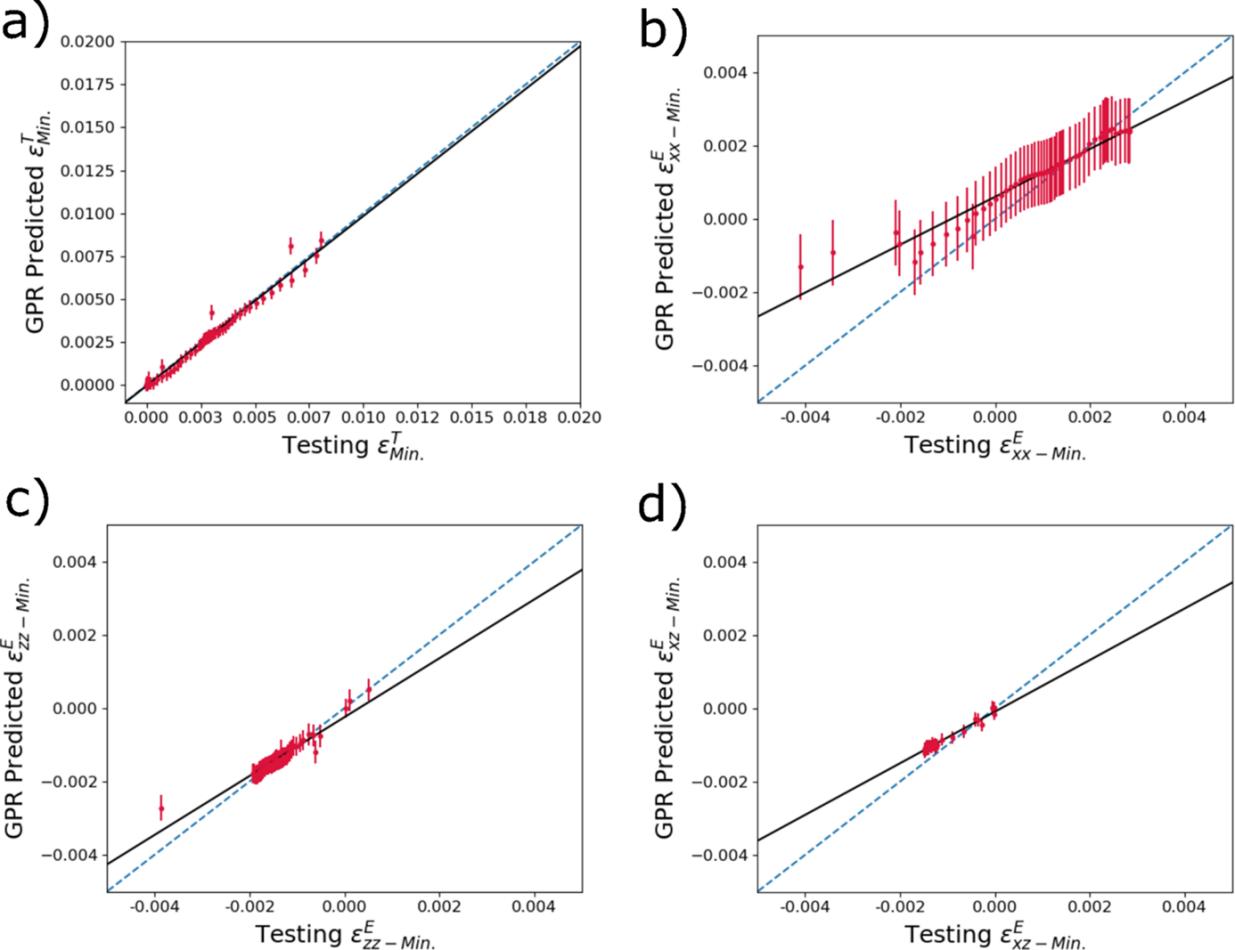
Accuracy predicting reserved testing data of trained surrogate models to determine the minimum of (*a*) 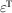, (*b*) 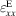, (*c*) 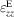 and (*d*) 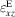 within a diffraction volume from input diffraction spectra. The dashed blue line denotes one-to-one correlation, and the black line is a linear fit.

Often of more interest are the maximum and minimum strains, particularly the maximum, within the diffraction volume. Fig. 8 ▸ shows comparisons between maximum strains in the reserved testing data and the various GPR model predictions. For 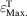 in Fig. 8 ▸(*a*), there is a small under-prediction of the maximum strain at higher strain (temperature values). This is similar to 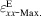 in Fig. 8 ▸(*b*) which also shows some degree of under-prediction at higher strain values. In Figs. 8 ▸(*c*) and 8 ▸(*d*), it can be seen that there is a small under-prediction of 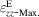 and 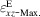, respectively, across the diffraction ranges of strain shown. The under-predictions across all strain components may be related to very small volume fractions of material in general contributing the diffraction peaks in comparison with the mean. Similarly to Fig. 8 ▸, Fig. 9 ▸ shows comparisons between minimum strains in the reserved testing data and the various GPR model predictions. The predictions of the minimum thermal strains 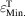 in Fig. 9 ▸(*a*) generally match well the ground truth across the total strain range. For the minimum 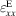 strains, the trends differ from predictions of the maximum in that there is a small over-prediction across the strain range. For 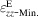 and 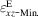, the strains are generally slightly under-predicted at high strain values and over-predicted at lower strain values, as seen in Figs. 9 ▸(*c*) and 9 ▸(*d*).

**Figure 10:**
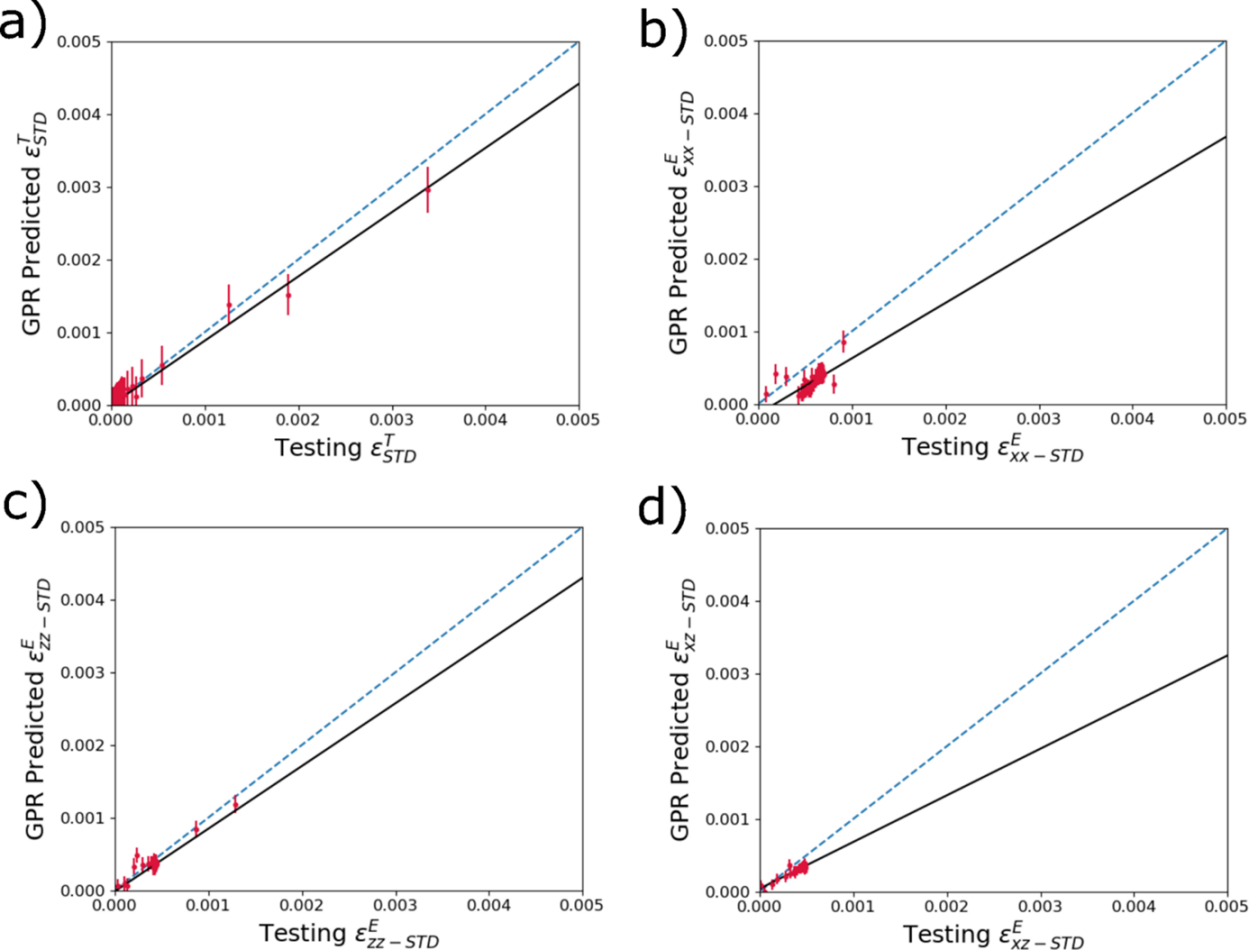
Accuracy predicting reserved testing data of trained surrogate models to determine the standard deviation of (*a*) 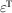, (*b*) 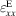, (*c*) 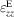 and (*d*) 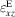 within a diffraction volume from input diffraction spectra. The dashed blue line denotes one-to-one correlation, and the black line is a linear fit.

The final strain metric for which GPR models were trained to extract from the diffraction data was the standard deviation (STD) of the distributions of strain within a diffraction volume. The results of the trained models for STD for 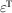, 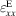, 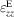 and 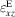 are shown in Figs. 10 ▸(*a*), 10 ▸(*b*), 10 ▸(*c*) and 10 ▸(*d*), respectively. The predictions of STD across GPR models across all strain types and components tend to under-predict the spread of the temperature distributions. This again is likely due to the very small contributions of extreme values (particularly maximums) which contribute very little intensity to the diffraction pattern. However, the GPR models are not predicting any aphysical STD values (such as a negative value).

## 5. Application to experimental data

**Figure 11:**
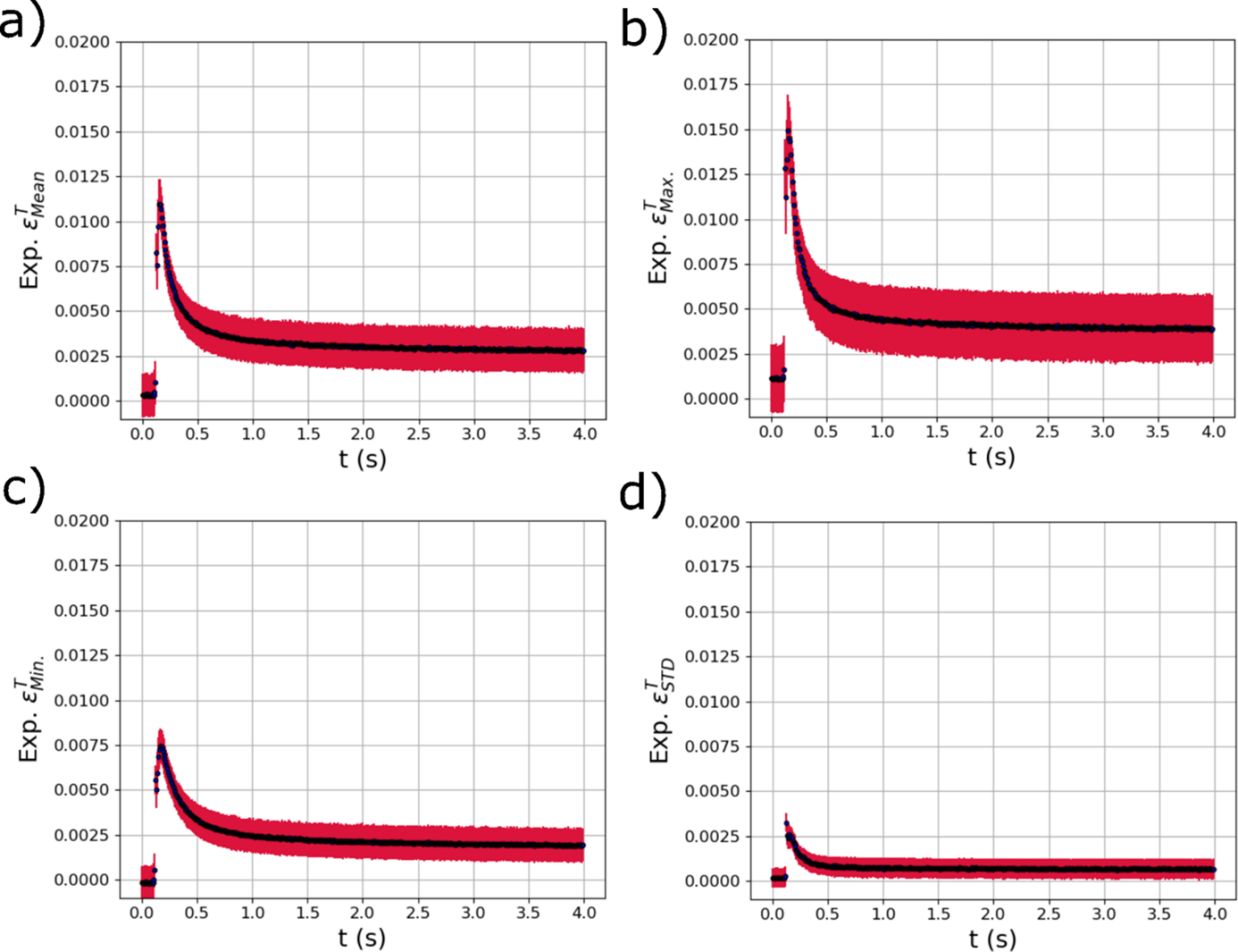
The evolving (*a*) mean 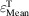, (*b*) maximum 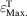, (*c*) minimum 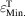 and (*d*) standard deviation 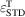 of the distribution of thermal strain within the experimental X-ray diffraction volume with respect to time *t* extracted using trained GPR surrogate models. The red error bars correspond to the square root of the variance (standard deviation) of the GPR surrogate model predictions.

The trained GPR surrogate models were used to analyze the evolution of the thermal and elastic strains within the diffraction volume during the *in situ* laser melting experiment described in Section 2. As mentioned, the raw experimental diffraction images were azimuthally binned into six different 1D line profiles that were concatenated into a single vector (Fig. 4 ▸). Results from applying the model to a single laser pass are presented; results from applying the trained surrogate models to a second laser pass at a different position on the same sample are provided in Appendix *A*[App appa] (Section *A*2). Fig. 11 ▸ shows the output metrics associated with thermal strains in the diffraction volume during the *in situ* experiment. The means of the GPR predictions from each diffraction measurement are shown with black dots, while the red error bars are the variance of the GPR predictions which can be used as a measure of uncertainty. The mean [Fig. 11 ▸(*a*)], maximum [Fig. 11 ▸(*b*)] and minimum [Fig. 11 ▸(*c*)] of the distribution of thermal strains all show a spike in thermal strain as the laser passes over the diffraction volume at 0.2 s. In addition, the STDs of the distributions [Fig. 11 ▸(*d*)] also rapidly increase as the laser passes over the specimen and then decrease back to near zero, which is expected as the entire specimen returns to room temperature.

**Figure 12:**
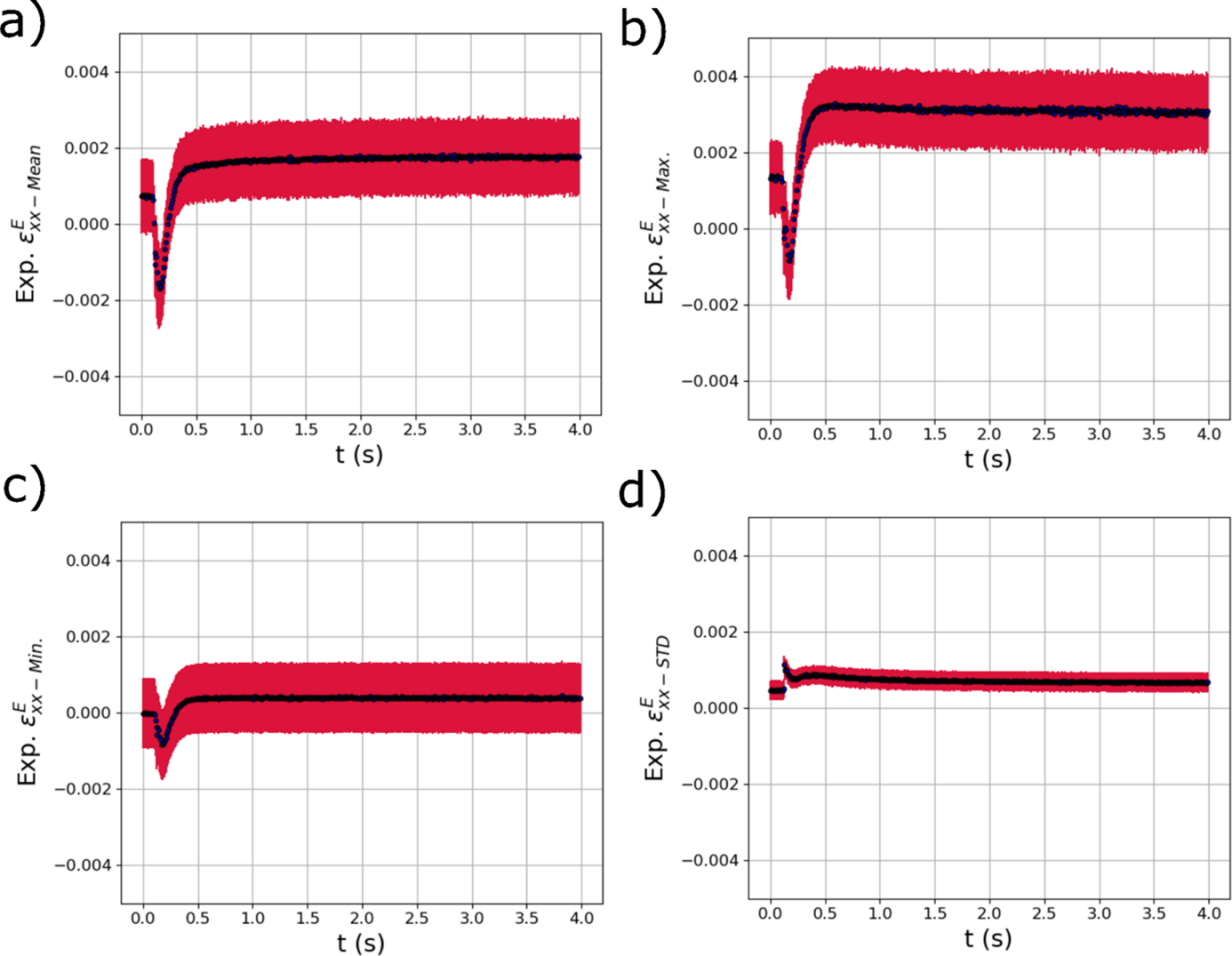
The evolving (*a*) mean 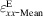, (*b*) maximum 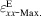, (*c*) minimum 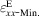 and (*d*) standard deviation 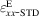 of the distribution of elastic strain within the experimental X-ray diffraction volume with respect to time *t* extracted using trained GPR surrogate models. The red error bars correspond to the square root of the variance (standard deviation) of the GPR surrogate model predictions.

Fig. 12 ▸ shows the output distribution metrics from the trained GPR models for 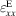. In the 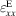 output metrics, there appears to be a small non-zero tensile strain at the beginning of the test (possibly from sample mounting or manufacture) which then becomes compressive as the laser passes over the sample. As the sample cools, the expected tensile residual stress develops with a larger magnitude than the original tensile strain at the beginning of the test. The general behavior of these strain distribution metrics output from the GPR model is consistent with the evolution of strains expected from simulation (see Fig. 6 ▸). The most critical observation is that the GPR surrogate models appear to be able to isolate the relatively small (in comparison with the thermal strains) compressive mechanical strains that occur due to localized heating, while simultaneously capturing the large thermal strains [Fig. 11 ▸(*a*)]. In addition, the STD of 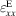 shown in Fig. 11 ▸(*d*) increased from the start of the test and did not decay as the temperature fell, reflecting the distributions of residual strain in the specimens.

**Figure 13:**
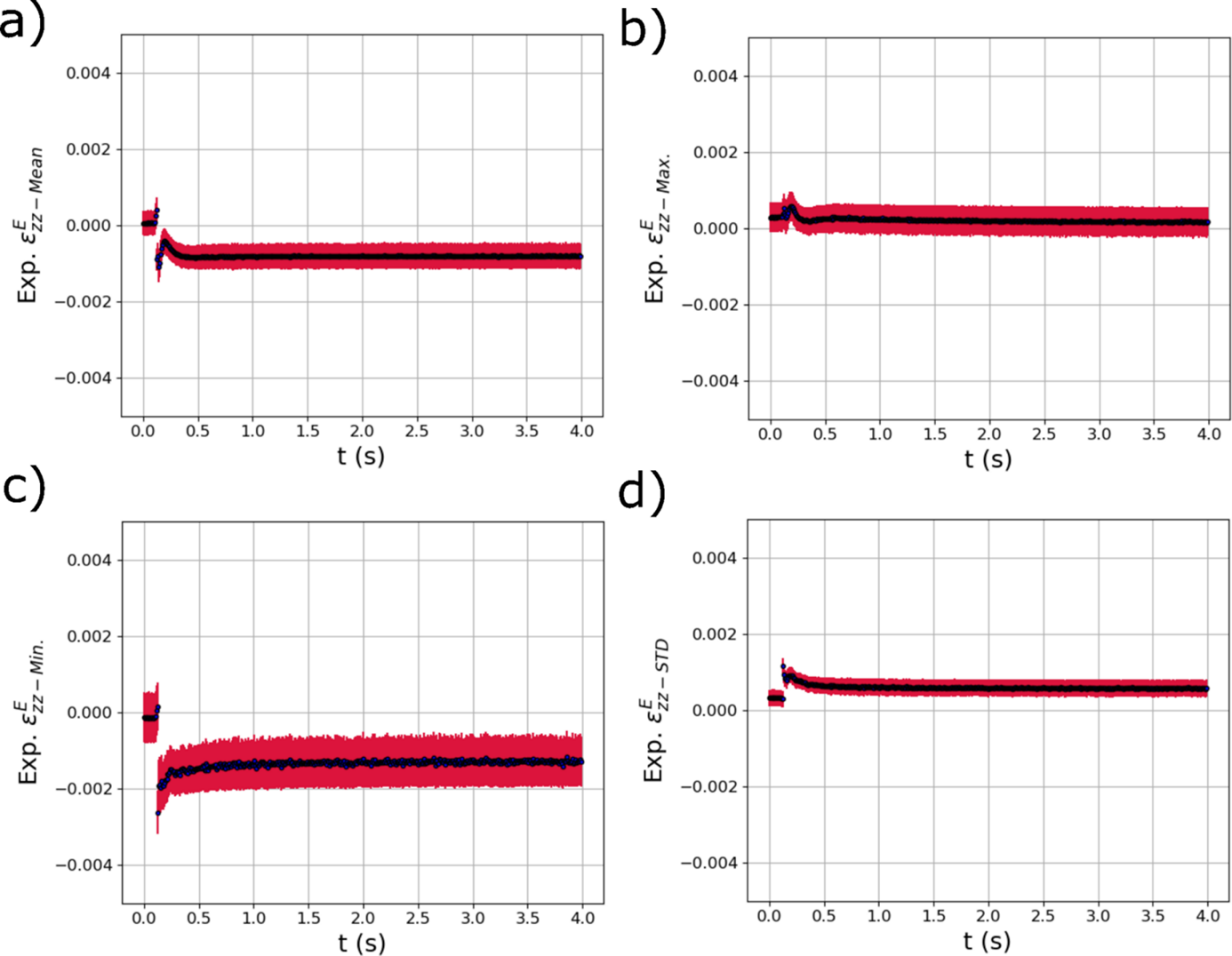
The evolving (*a*) mean 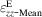, (*b*) maximum 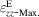, (*c*) minimum 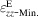 and (*d*) standard deviation 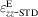 of the distribution of elastic strain within the experimental X-ray diffraction volume with respect to time *t* extracted using trained GPR surrogate models. The red error bars correspond to the square root of the variance (standard deviation) of the GPR surrogate model predictions.

**Figure 14:**
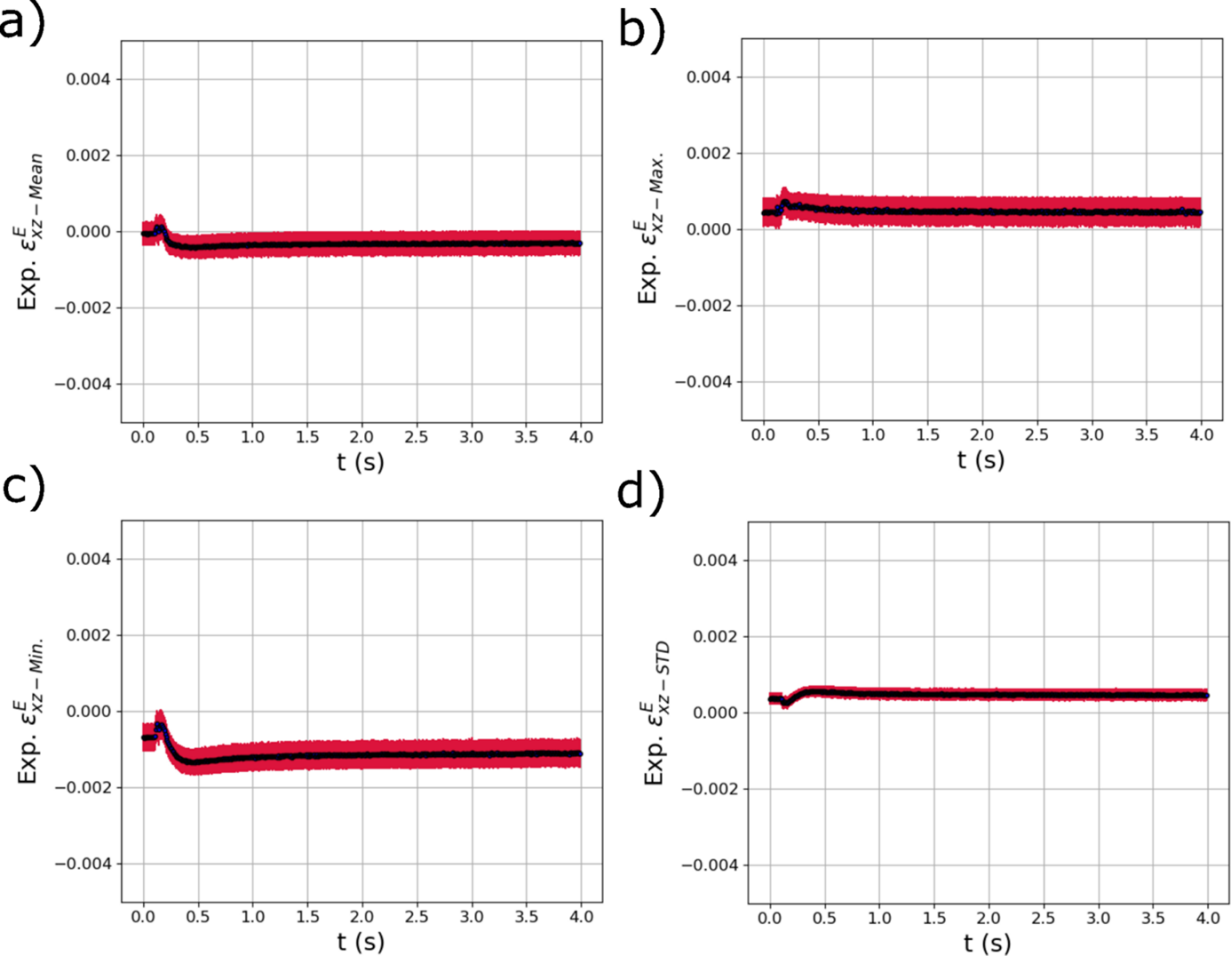
The evolving (*a*) mean 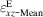, (*b*) maximum 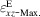, (*c*) minimum 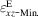 and (*d*) standard deviation 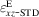 of the distribution of elastic strain within the experimental X-ray diffraction volume with respect to time *t* extracted using trained GPR surrogate models. The red error bars correspond to the square root of the variance (standard deviation) of the GPR surrogate model predictions.

The GPR model distribution metric outputs from the other two strain components, 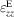 and 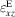, are shown in Fig. 13 ▸ and Fig. 14 ▸. In the distributions in Fig. 13 ▸, a difference of behavior is observed between the maximum [Fig. 13 ▸(*b*)] and minimum strains [Fig. 13 ▸(*c*)] in the distribution. While the maximum strains become slightly tensile as the laser passes over the specimen, the minimum strains become negative and then remain negative through cooling. The final mean of the distribution of 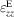 is consistent with unconstrained Poisson contraction from the tensile residual strains that developed. Similar to 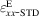, 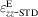 peaks as the laser passes over the sample then decays to a value marginally larger than at the start of the test [Fig. 14 ▸(*d*)]. The distribution metrics of 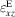 generally show similar trends to those of 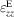 as can be see in Fig. 14 ▸. However, the STD of the distribution of shear strains 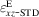 is at a minimum as the laser passes over the diffraction volume.

## 6. Discussion

Here, we demonstrated a machine-learning-enabled approach to analyzing complex diffraction patterns from volumes containing distributions of thermal and mechanical strains. Future application of this approach is to support the development of new classes of process diagnostics for (additive) manufacturing. Porosity from lack-of-fusion or keyholing and residual stress from unexpected process excursions are still challenges leading to failed builds. Surface optical measurements and thermography provide some insight into porosity formation, but in particular do not provide insight into residual stress development. While not immediate, rapid advances in high-brightness laboratory X-ray source technology [*e.g.* current metal jet (Larsson *et al.*, 2011 ▸) and future thin-film diamond sources (Kandlakunta *et al.*, 2019 ▸; Tan *et al.*, 2022 ▸)] could lead to in-chamber AM diagnostics. To characterize the thermomechanical state of the material in-chamber using these sources, non-standard analysis of diffraction data is needed. As opposed to trying to determine what the average material response is under relatively well characterized thermo­mechanical loading, instead, a process diagnostic needs to quantify thermomechanical state using relatively well understood material response (which is the approach taken here). While our approach was applied to analyzing material being laser melted, it can also be applied to any rapid, complex testing scenario where distributions of thermal and mechanical strains are expected (*e.g.* dynamic loading). In addition, our approach provides a path forward for analyzing other types of challenging material processing scenarios where measured lattice deformation is composed of different types of eigenstrains besides temperature (*e.g.* intrinsic piezoelectric or chemical).

Using the ML surrogate model trained with thermomechanical simulations for analyzing the diffraction data provides several primary benefits over more standard methods that analyze the shifts of diffraction peaks using analytical function fitting to probe temperature (Hocine *et al.*, 2020 ▸; Oh *et al.*, 2021*a* ▸; Oh *et al.*, 2021*b* ▸) or temperature and mechanical strain (Schmeiser *et al.*, 2021 ▸; Scheel *et al.*, 2023 ▸). First, the lack of peak fitting allows a wider range of peak shapes to be quantitatively analyzed. For example, ‘split’ diffraction peaks are often encountered due to the wide differences, or distributions, of temperatures between the inside and outside of a melted region. Second, the thermomechanical modeling provides a means of decoupling thermal strains and volumetric elastic strains which will deform the lattice in the same fashion. Third, the method provides metrics about the distributions of strains present which can be of significant interest for microstructural evolution as many features develop as functions of field gradients. Lastly, the GPR surrogate models provide a natural measure of uncertainty through output of the variance of the model predictions.

As was seen in Section 4, the GPR models were most accurate at predicting the mean of the thermal and elastic strain distributions present in the reserved testing data. This is not surprising as the mean of the distribution also contributes to the peak of diffracted intensity. Importantly, the GPR models appear to be effective at fully deconvoluting thermal and elastic strains which, again, is a challenge due to the inability to directly differentiate between thermal and volumetric elastic strains in cubic materials; applying the GPR models to the *in situ* experimental data yielded evolution of thermal and elastic strains that was consistent with physics-based modeling.

While still generally accurate, the predictions of maximums and minimums of the various strain distributions were subject to under- or over-predictions, usually at the extremes of the ranges of strain used for model training. The predictions of standard deviations of strain distributions were also generally under-predicted. Both of these observations are likely due to the fact that volumes of material with extreme values of strain generally do not contribute to the primary body of diffraction peaks and these extreme thermomechanical states are also extremely transient and as such have a small presence in the simulated training data. Further effort is required to determine if extreme value accuracy could be improved by increasing the number of simulations and the amount of data used for surrogate model training. In particular, increasing the number of microstructural configurations used for GPR model training may be beneficial (*i.e.* instantiating the same thermo­mechanical distributions with different grain orientation configurations).

For this effort, we took the approach of performing high-fidelity simulations for both generating thermal (finite difference heat transfer and fluid flow) and elastic (finite element elasto-plasticity) strain distributions. The rationale of this approach was that by training with high-fidelity thermal and elastic strain fields that may be present, less training data would be necessary to analyze experimental data from similar conditions. This approach appears to be successful, providing results from experimental data in line with what is expected, but the downside is that the complete modeling of the physical melting and cooling process is challenging. To lower the modeling burden, an open question worth investigating is what fidelity of thermomechanical simulations is necessary for sufficiently accurate thermomechanical quantification from experimental measurements? Another approach may be to train the models with lower computational burden semi-analytical models (Weisz-Patrault, 2020 ▸) or high volumes of low-fidelity simulation data. This would ease the accessibility and adoption of our proposed approach. Similarly, another avenue that requires further investigation is the sensitivity of the GPR model predictions to microstructural and material parameters used in the thermomechanical modeling for GPR training. While the GPR models are effectively connecting thermal and elastic strain distributions with diffracted intensity distributions, microstructural and material parameters will alter the space of distributions for model training, which may alter final predictions. In this work, the morphologies and size distributions of grains are not a perfect match to experiment, but the GPR model still produces rational results indicating that it may be possible to relax model accuracy. Furthermore, here we opted for use of accurate material parameters, specifically those generated with first-principles DFT. However, this process is intensive and our presented approach would be significantly eased if literature values with less provenance or similar alloy compositions (*e.g.* using pure Ni properties for a superalloy) could achieve similar prediction results.

## 7. Summary

High-fidelity atomistic, heat transfer and fluid flow, thermomechanical and X-ray scattering simulations were brought together to train machine-learning (Gaussian process regression, GPR) surrogate models for analyzing diffraction data with complex thermomechanical distributions of deformation present. The trained GPR models were then transferred into the experimental domain to extract thermal and elastic strain distribution metrics from *in situ* diffraction data gathered during laser melting of IN625. In summary:

(i) The physical modeling was used to generate over 2000 diffraction images with various thermal, elastic and microstructural configurations for GPR model training.

(ii) The GPR models are capable of separating the effects of thermal and mechanical elastic strain distributions, including at high temperatures where the magnitude of thermal strains is significantly larger.

(iii) The GPR models were most accurate at predicting the mean of the thermal and elastic strain distributions from diffraction data, followed by the maximums and minimums of the distribution. In general, the GPR models tended to under-predict the standard deviation (or spread) of the thermal and elastic strains present.

## 8. Data and code availability statement

All data used for this work are available upon reasonable request. The Python-based diffraction simulation and GPR training codes are also available upon request.

## Figures and Tables

**Figure 15:**
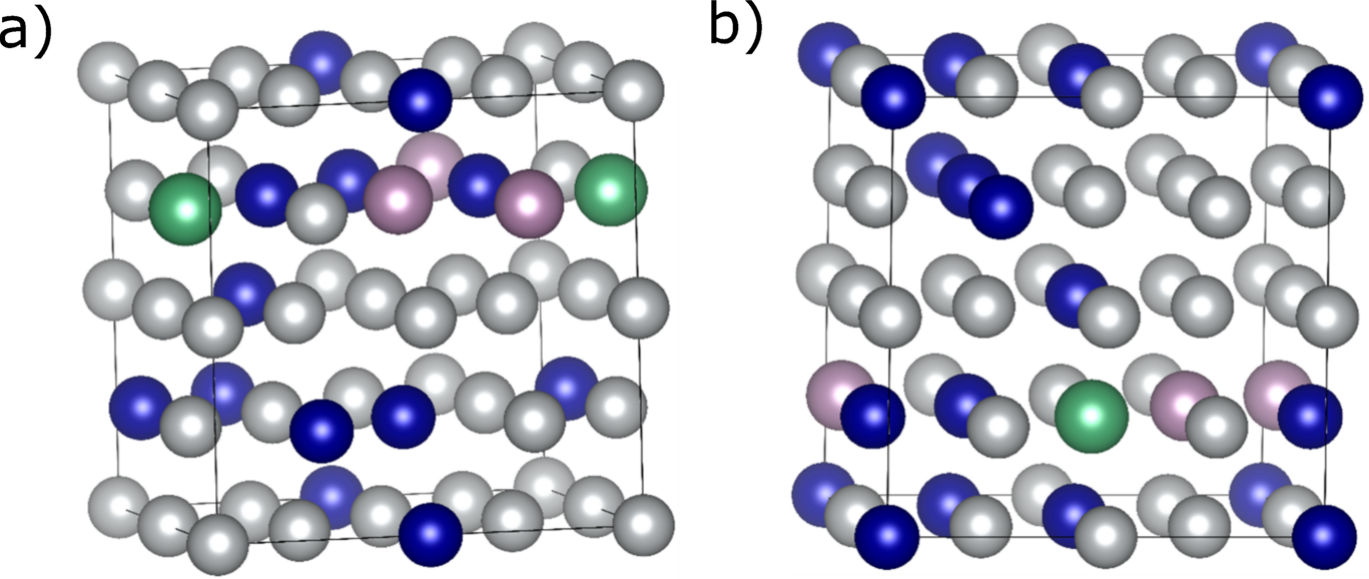
(*a*) One of the generated SQSs and (*b*) one of the generated SCRAPs of Ni_21_Cr_8_Mo_2_Nb_1_ (32-atom supercell), where the colored spheres represent different atom types: Ni (gray), Cr (blue), Mo (purple) and Nb (green).

**Figure 16:**
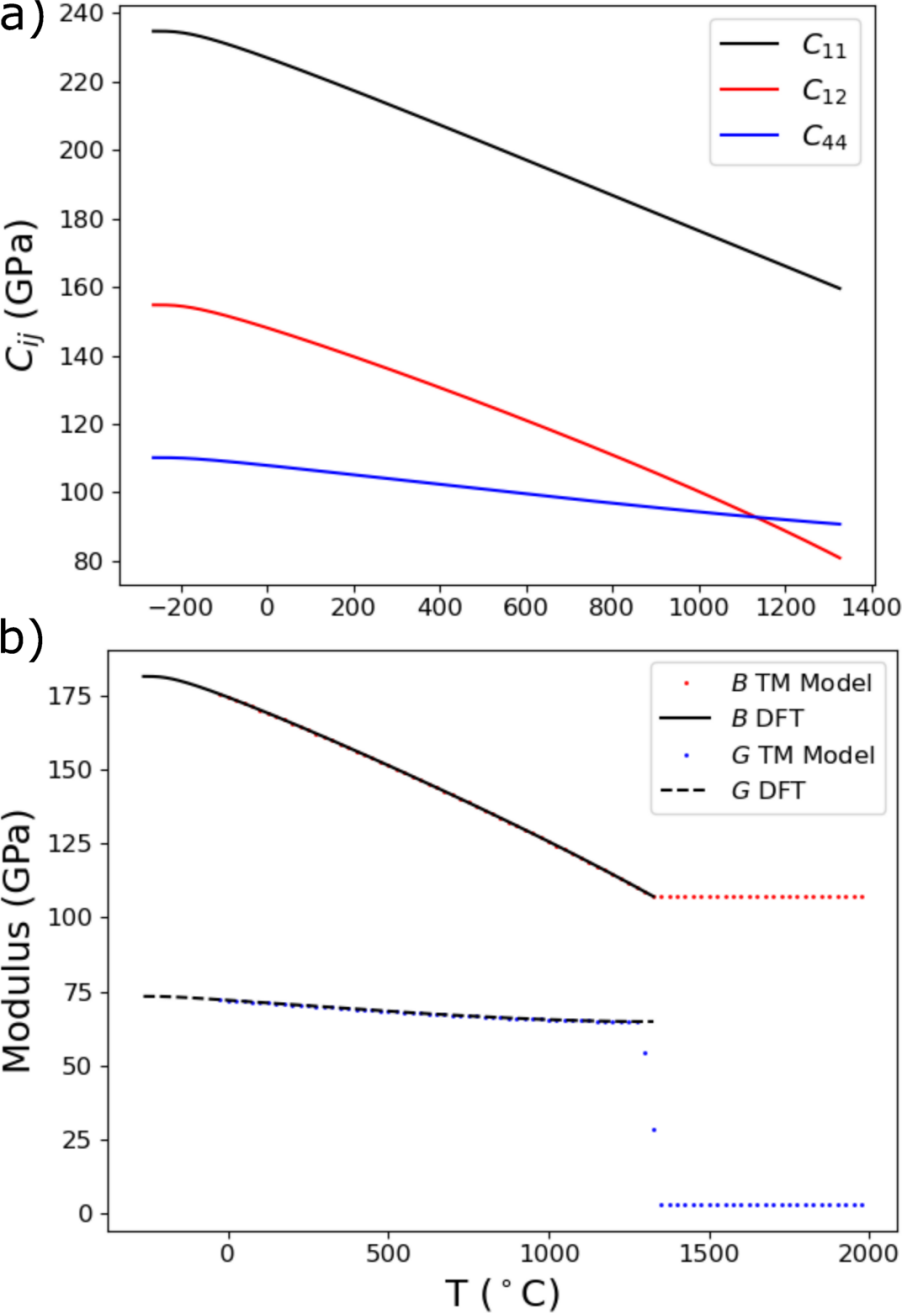
(*a*) Temperature-dependent cubic single-crystal elastic moduli 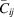 of AM IN625 calculated using DFT-based quasi-harmonic analysis (QHA) and quasi-static analysis (QSA). (*b*) Bulk modulus *B* (black line) and shear modulus *G* (black dashed line) calculated from the DFT single-crystal elastic moduli. Also shown are the bulk modulus *B* (red dots) and shear modulus *G* (blue dots) used as input for the thermomechanical model (TM model).

**Figure 17:**
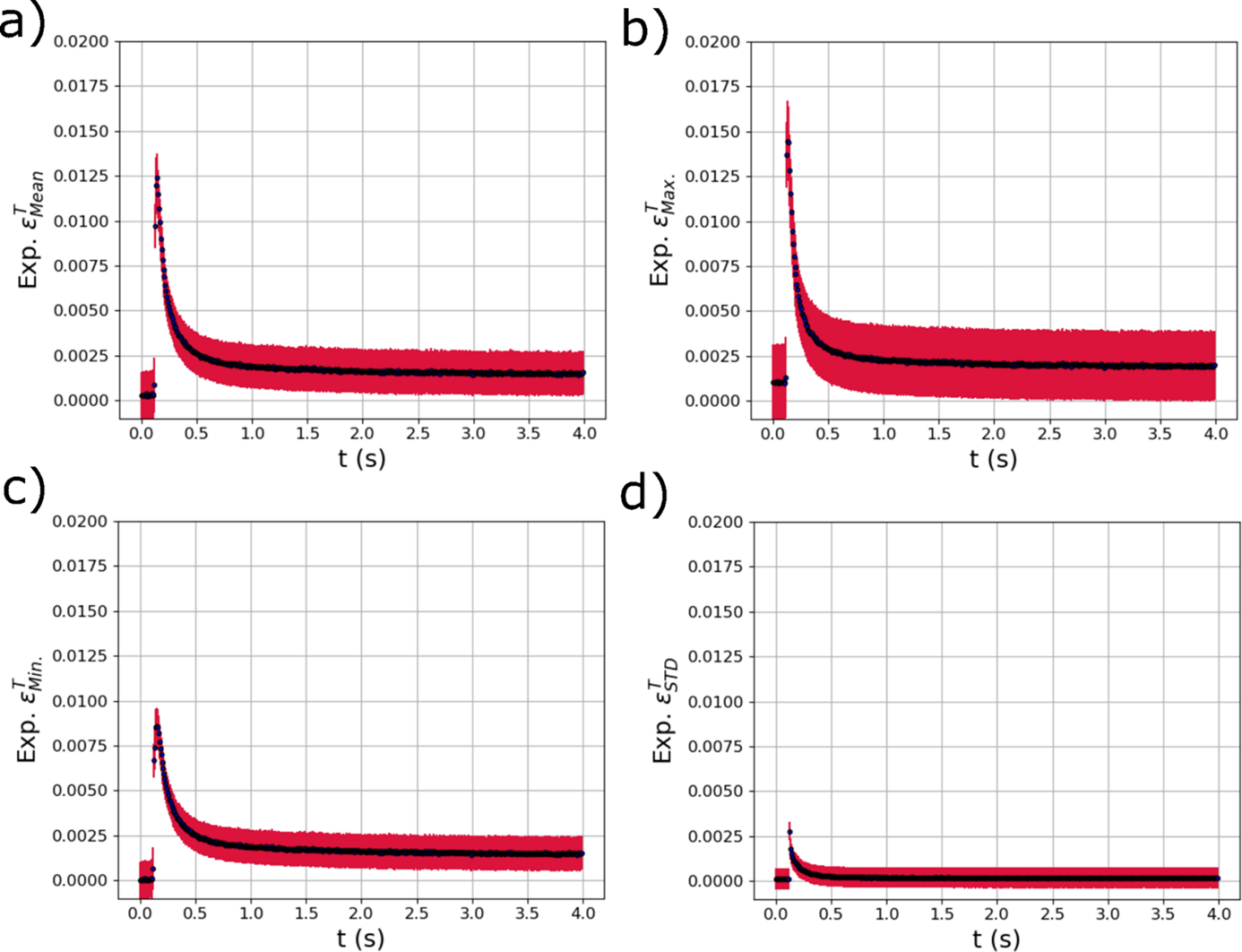
The evolving (*a*) mean 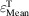, (*b*) maximum 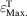, (*c*) minimum 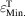 and (*d*) standard deviation 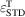 of the distribution of thermal strain within the duplicate experimental X-ray diffraction volume with respect to time *t* extracted using trained GPR surrogate models. The red error bars correspond to the square root of the variance (standard deviation) of the GPR surrogate model predictions.

**Figure 18:**
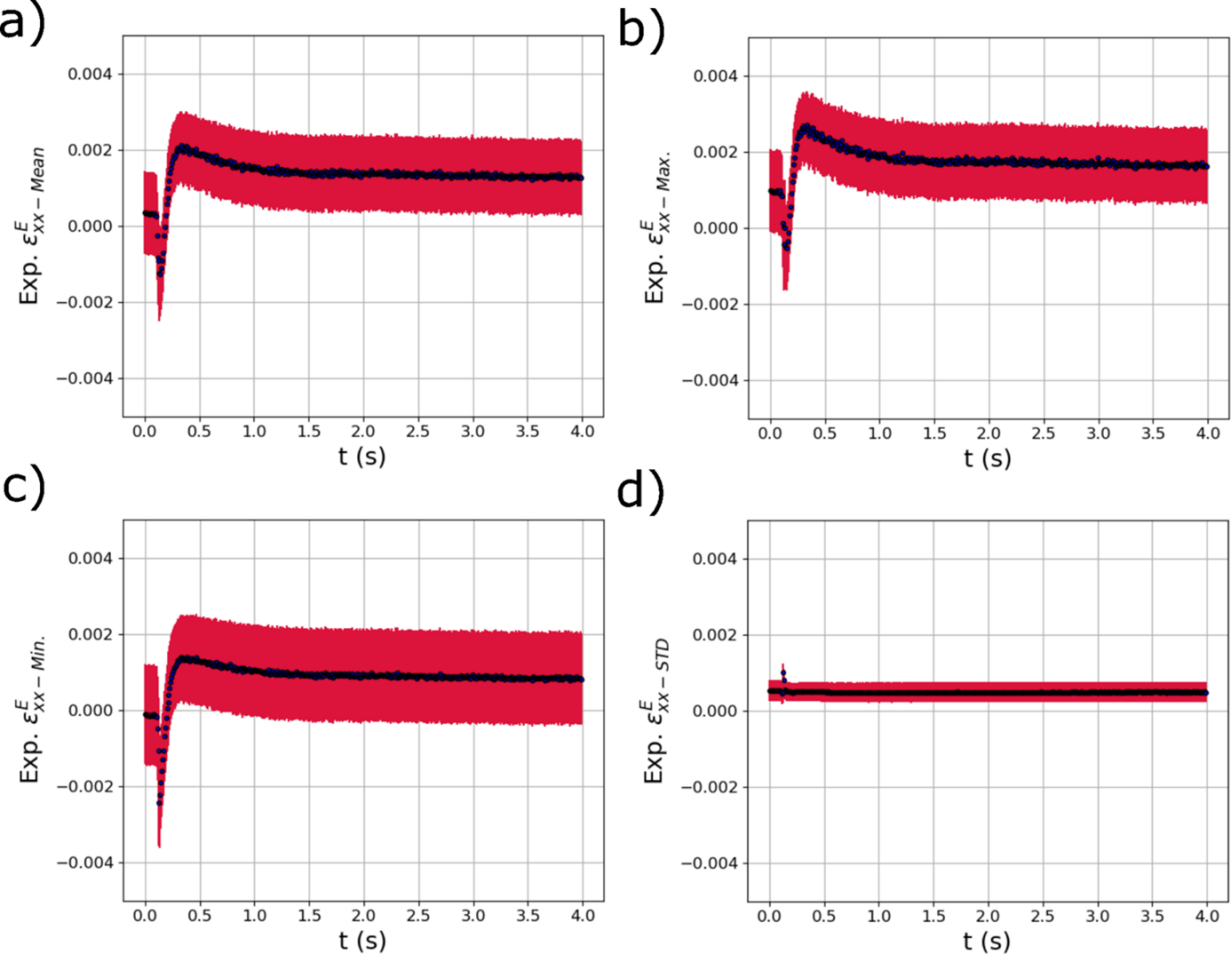
The evolving (*a*) mean 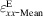, (*b*) maximum 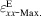, (*c*) minimum 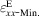 and (*d*) standard deviation 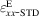 of the distribution of elastic strain within the duplicate experimental X-ray diffraction volume with respect to time *t* extracted using trained GPR surrogate models. The red error bars correspond to the square root of the variance (standard deviation) of the GPR surrogate model predictions.

**Figure 19:**
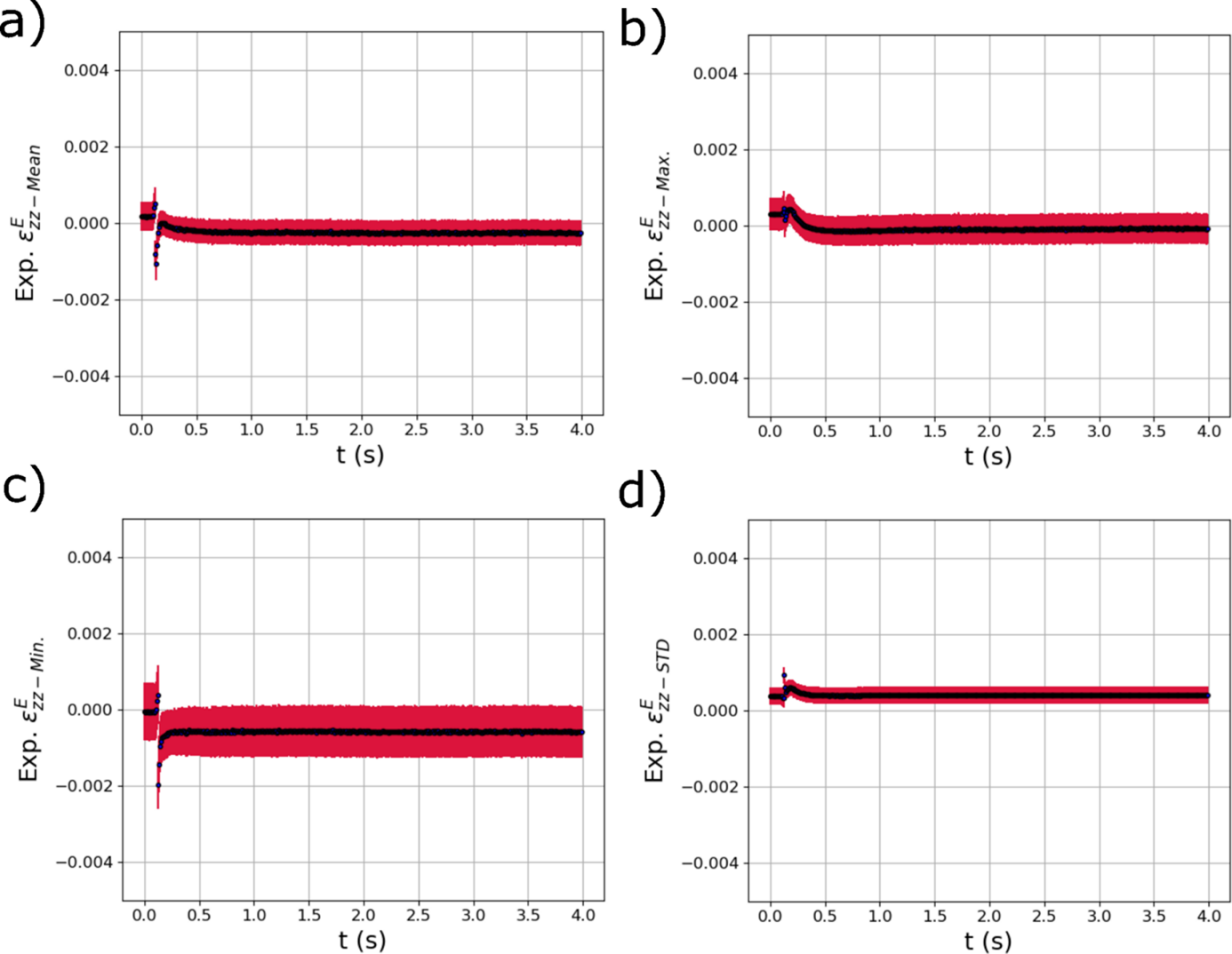
The evolving (*a*) mean 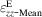, (*b*) maximum 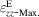, (*c*) minimum 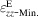 and (*d*) standard deviation 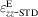 of the distribution of elastic strain within the duplicate experimental X-ray diffraction volume with respect to time *t* extracted using trained GPR surrogate models. The red error bars correspond to the square root of the variance (standard deviation) of the GPR surrogate model predictions.

**Figure 20:**
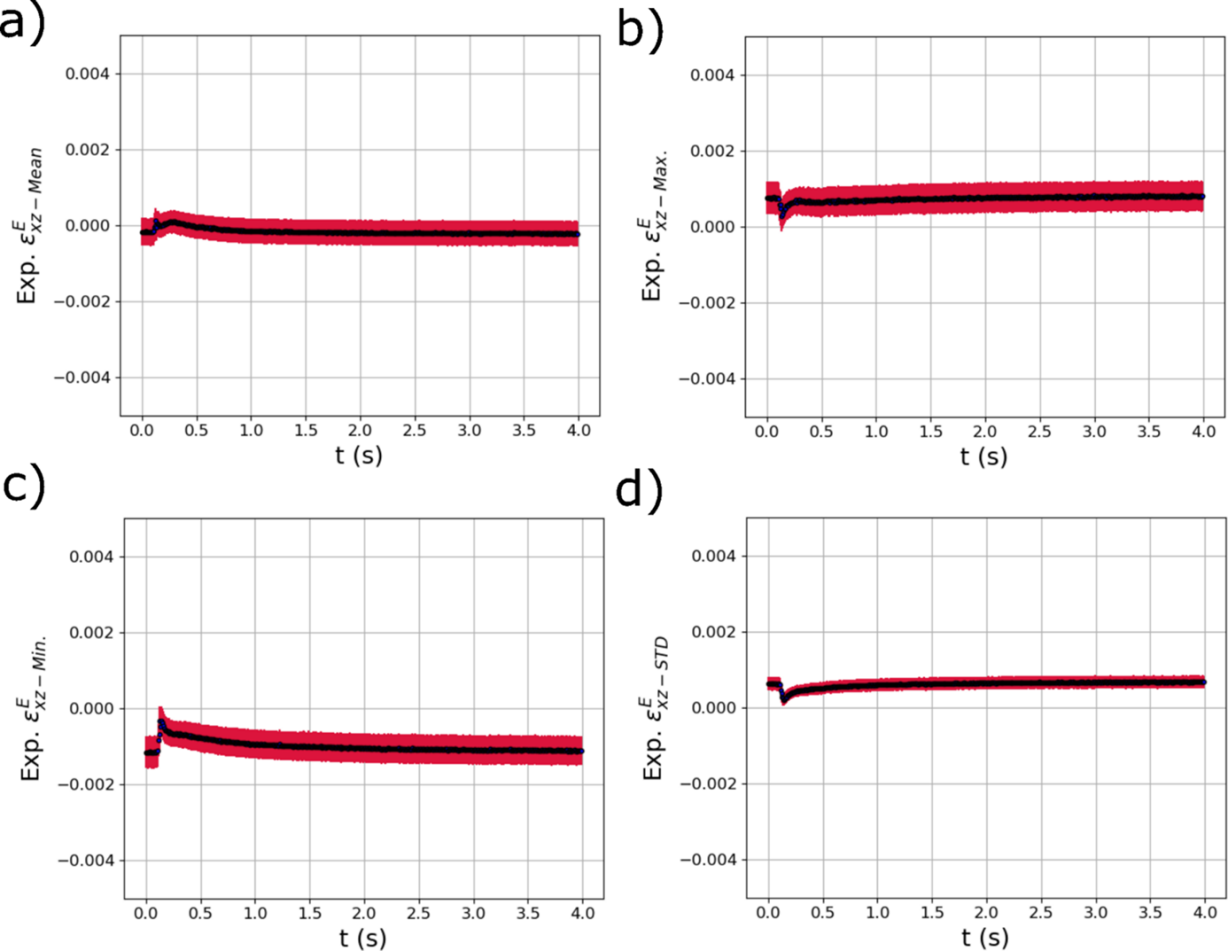
The evolving (*a*) mean 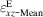, (*b*) maximum 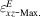, (*c*) minimum 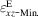 and (*d*) standard deviation 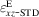 of the distribution of elastic strain within the duplicate experimental X-ray diffraction volume with respect to time *t* extracted using trained GPR surrogate models. The red error bars correspond to the square root of the variance (standard deviation) of the GPR surrogate model predictions.

**Table 1:** IN625 model parameters used in the heat transfer and fluid flow modeling calculated using *JMatPro* Thermal conductivity and specific heat are temperature *T* dependent (units K).

Model parameter	Value
Density (kg m^−3^)	8440
Solidus temperature (K)	1563
Liquidus temperature (K)	1623
Specific heat (J/kg/K)	360.4 + 0.26*T* − 4 × 10^−6^ *T*^2^
Thermal conductivity (W/m/K)	0.56 + 2.9 × 10^−2^*T* − 7 × 10^−6^ *T*^2^
Latent heat of fusion (J kg^−1^)	209.2 × 10^3^
Viscosity (kg/m/s)	5.3 × 10^−3^
Temperature coefficient of surface tension (N/m/K)	−0.37 × 10^−3^
Surface tension (N m^−1^)	1.82
Absorptivity factor	0.3
Emissivity factor	0.4

**Table 2:** Table summarizing the data sets generated for GPR Thermomechanical simulations were performed with different laser powers and velocities, in addition to X-ray simulations moving the position of the X-ray beam with respect to the top of the sample.

No.	Power (W)	Velocity (m s^−1^)	X-ray position (µm)
1	100	0.04	20
2	100	0.04	40
3	100	0.04	60
4	100	0.05	20
5	100	0.05	40
6	100	0.05	60
7	100	0.06	20
8	100	0.06	40
9	100	0.06	60
10	120	0.04	20
11	120	0.04	40
12	120	0.04	60
13	120	0.05	20
14	120	0.05	40
15	120	0.05	60
16	120	0.06	20
17	120	0.06	40
18	120	0.06	60
19	140	0.04	20
20	140	0.04	40
21	140	0.04	60
22	140	0.05	20
23	140	0.05	40
24	140	0.05	60
25	140	0.06	20
26	140	0.06	40
27	140	0.06	60

**Table 3:** Temperature-dependent yield σ^Y^(*T*) and hardening *H*(*T*) parameters used in the elasto-plasticity simulations

Temperature (°C)	σ^Y^(*T*) (MPa)	*H*(*T*) (MPa)
25	691	395
500	615	395
816	260	286
982	105	35
1093	57	20

## Appendix A

### A1. First-principles thermomechanical property calculations

We utilized first-principles DFT using the *VASP* code (Kresse & Hafner, 1993 ▸) to generate temperature-dependent IN625 elastic moduli and coefficients of thermal expansion for input into later thermomechanical modeling. Our process has been described in detail elsewhere (Shang *et al.*, 2010 ▸; Shang *et al.*, 2024 ▸), and is summarized here. In this approach, the Helmholtz energy *F* has a 0 K static energy contribution, a vibrational contribution and a thermal electron contribution. Here, each individual contribution to the Helmholtz energy is predicted independently, with the vibrational and thermal electron contributions calculated as a function of volume *V* at a given temperature *T*, and then the three contributions summed. From the temperature-dependent Helmholtz energy, the equilibrium volume  is calculated as a function of temperature. The linear coefficient of thermal expansion α is then calculated as

For the IN625 property modeling, we used the compositions reported for AM Bench 2018 material (Levine *et al.*, 2020 ▸) of which the same powder was used here. In the IN625, the major alloying components besides Ni are Cr (20.61 wt%), Nb (3.97 wt%) and Mo (8.82 wt%). Based on CALPHAD predictions, there are two major phases, *i.e.* the dominant face-centered cubic (f.c.c.) matrix (γ) and the topologically close-packed (t.c.p.) phase, δ. Here, we assume that the thermal expansion and elastic properties are primarily dependent on the f.c.c. matrix. The composition of the f.c.c. matrix according to CALPHAD predictions is roughly Ni_21_Cr_8_Mo_2_Nb_1_ (Shang *et al.*, 2024 ▸). To predict properties, relatively small cubic supercells (32-atom) were modeled, and then the results were averaged to strike a balance between accuracy and computational efficiency. We generated six special quasirandom structures (SQSs) (Zunger *et al.*, 1990 ▸) and six supercells in random approximates (SCRAPs) (Singh *et al.*, 2021 ▸). Fig. 15 ▸ shows representative SQS and SCRAPs structures, with Fig. 15 ▸(*a*) showing one of the generated SQSs and Fig. 15 ▸(*b*) one of the generated SCRAPs. Temperature-dependent Helmholtz energy was calculated for each of these supercells and then used for both coefficient of thermal expansion and elastic moduli calculations [see details including first-principles settings in the work of Shang *et al.* (2024 ▸)]. Fig. 5 ▸(*a*) shows the calculated temperature-dependent coefficient of thermal expansion  using this procedure. The values matched well with experimental data used by Lim *et al.* (2023 ▸) within the temperature bounds measured.

Single-crystal elastic moduli () as a function of temperature *T* were predicted using the quasi-static approach (QSA) (Wang *et al.*, 2010 ▸; Shang *et al.*, 2010 ▸; Shang *et al.*, 2012 ▸) according to the predicted relationship between  and *V* at 0 K and the previously calculated relationship between *T* and *V* (*i.e.* thermal expansion) (Shang *et al.*, 2024 ▸), resulting in the predicted . Note that the DFT-based calculations of  at 0 K were predicted using the anisotropic form of Hooke’s law (Shang *et al.*, 2007 ▸), where the employed non-zero strains applied are ±0.01. Since both the SQSs and SCRAPs of IN625 are monoclinic configurations, averaged values of the elastic moduli were used to calculate cubic elastic moduli , *i.e.*,  and . Note that the averaged  values usually have deviations less than 10% from the mean for each monoclinic configuration, such as  255.8 GPa,  262.2 GPa and  271.7 GPa for a SCRAPS configuration (SCRAPS_*f*_) at its equilibrium volume. Fig. 16 ▸(*a*) shows the cubic single-crystal moduli ,  and  determined from the DFT calculations. From the figure, we see that both  and  decrease faster than  with increasing temperature.

For input into the continuum thermomechanical model (Section 3.2), ,  and  were used to generate isotropic elastic moduli. The means of the Reuss and Voigt averages of an untextured cubic polycrystal (Hosford, 1993 ▸) were used to calculate Young’s modulus  and the shear modulus , from which Poisson’s ratio  was calculated. For temperatures beyond the melting temperature of the material, the bulk modulus  of the material was assumed to remain constant while the shear modulus was reduced to 5% of the value prior to melting. The resulting bulk modulus and shear modulus calculated from this process are provided in Fig. 16 ▸(*b*).

### A2. Duplicate experimental analysis

After the initial laser pass over the wall specimen from which results were presented in Section 5, the specimen was allowed to nominally cool and then was translated approximately 2 mm with respect to the laser and incoming X-ray beam along **x** such that unmelted material was now illuminated. The previously described *in situ* laser melting experiment was then repeated with the same X-ray beam, detector and laser parameters. The diffraction images from this measurement were then processed in an identical manner to that described previously and used as input for the trained GPR models. Fig. 17 ▸ shows the predicted  metrics, Fig. 18 ▸ shows  metrics, Fig. 19 ▸ shows  metrics and Fig. 20 ▸ shows  metrics. As can be seen, the evolution of the ,  and  is very similar to that reported in Section 5, providing confidence in the repeatability of the measurements and data analysis approach. There are differences in the distribution metrics output for , particularly as the laser passed over the diffraction volume, but this is likely related to the shear strains being the most sensitive to any minor misalignment of the laser path with the wall specimen geometry.
